# Commensal bacteria and essential amino acids control food choice behavior and reproduction

**DOI:** 10.1371/journal.pbio.2000862

**Published:** 2017-04-25

**Authors:** Ricardo Leitão-Gonçalves, Zita Carvalho-Santos, Ana Patrícia Francisco, Gabriela Tondolo Fioreze, Margarida Anjos, Célia Baltazar, Ana Paula Elias, Pavel M. Itskov, Matthew D. W. Piper, Carlos Ribeiro

**Affiliations:** 1 Behavior and Metabolism Laboratory, Champalimaud Neuroscience Programme, Champalimaud Centre for the Unknown, Lisbon, Portugal; 2 School of Biological Sciences, Monash University, Clayton, Victoria, Australia; Rockefeller University, United States of America

## Abstract

Choosing the right nutrients to consume is essential to health and wellbeing across species. However, the factors that influence these decisions are poorly understood. This is particularly true for dietary proteins, which are important determinants of lifespan and reproduction. We show that in *Drosophila melanogaster*, essential amino acids (eAAs) and the concerted action of the commensal bacteria *Acetobacter pomorum* and *Lactobacilli* are critical modulators of food choice. Using a chemically defined diet, we show that the absence of any single eAA from the diet is sufficient to elicit specific appetites for amino acid (AA)-rich food. Furthermore, commensal bacteria buffer the animal from the lack of dietary eAAs: both increased yeast appetite and decreased reproduction induced by eAA deprivation are rescued by the presence of commensals. Surprisingly, these effects do not seem to be due to changes in AA titers, suggesting that gut bacteria act through a different mechanism to change behavior and reproduction. Thus, eAAs and commensal bacteria are potent modulators of feeding decisions and reproductive output. This demonstrates how the interaction of specific nutrients with the microbiome can shape behavioral decisions and life history traits.

## Author summary

What animals, including humans, choose to eat has a tremendous impact on health and wellbeing. Though intake of dietary proteins and amino acids is essential for animals, excessive consumption of these nutrients is known to have detrimental effects. Many animals, therefore, execute precise control over the intake of these key nutrients. However, the factors controlling protein appetite are poorly understood. Here, we show that in the vinegar fly *Drosophila melanogaster*, essential amino acids and gut bacteria are key modulators of protein appetite. Lack of any one essential amino acid from the diet produces a strong and specific appetite for proteinaceous or amino acid–rich food. However, flies with an appropriate microbiome do not develop this protein appetite. Specifically, two gut bacteria species, *Acetobacter pomorum* and *Lactobacilli*, work together to suppress protein appetite. Furthermore, we show that flies lacking dietary essential amino acids have reduced reproductive output, an effect which is also rescued by gut bacteria. Finally, based on metabolite measurements, we propose that the influence of bacteria on host physiology and behavior is not mediated by changing amino acid levels. Our study demonstrates how the interaction of specific nutrients with the microbiome can shape behavior and animal fitness and suggests that they do so through a novel mechanism.

## Introduction

The appropriate intake of nutrients has a major beneficial impact on health and lifespan [1–3]. The level of dietary protein intake has emerged as a key determinant of overall mortality, fecundity, and lifespan in species ranging from humans [4] to mice [5] and *Drosophila* [6–9]. Accordingly, animals, including humans, are able to direct food choice in order to tightly control protein intake [2,3,10–13]. Despite the striking physiological and behavioral impact of nutritional proteins, how animals direct feeding decisions to ensure protein homeostasis is not understood. A major obstacle in identifying the rules governing food choice is the nutritional complexity of natural foods, which hinders the discovery of the nutritional variables controlling feeding decisions.

In *Drosophila melanogaster*, yeast is thought to cover the protein as well as most other noncaloric nutritional requirements [7]. In adult females, yeast appetite is driven by two main internal states: mating and lack of yeast [12–15]. The molecular and circuit mechanisms leading to an increase in yeast appetite upon mating have been extensively characterized. During copulation, the male-derived Sex Peptide is transferred to the female and acts on the neuronal Sex Peptide Receptor, leading to the silencing of a postmating neuronal circuit, consisting of SPSN/SAG/octopamine components, which projects to the central brain to change feeding preference from sugar to yeast [12,14,16]. Besides mating, the other known determinant of protein intake is removal of yeast from the diet, which leads to a strong compensatory appetite for yeast [12]. The mechanisms underlying this homeostatic change in appetite are less well understood. This is partially due to the fact that yeast is a complex food containing different nutrients, including amino acids (AAs), carbohydrates, vitamins, and sterols [17,18]. However, it is still unknown which nutrient(s), when absent, triggers flies to ingest yeast. Identifying the mechanisms controlling protein homeostasis in *Drosophila* requires untangling this nutritional complexity.

The interaction of microbiota with ingested nutrients has emerged as a major determinant of health and disease, including obesity [19–24]. Commensal bacteria have also been proposed to affect a wide array of brain functions [25–29] ranging from bulk food intake [30] to anxiety [31–33], neurodevelopmental disorders [34], and social behavior [35]. Despite being an intense field of research, the importance of microbe–nutrient interactions in influencing behavior remains poorly understood. In vertebrates, this task is especially challenging given the complexity of their microbiota and the large set of nutritional parameters that could influence their function. Furthermore, in the context of nutrition, research on microbiota has mainly focused on their role in carbohydrate homeostasis [21,36]. More recently, however, the importance of commensal bacteria in controlling growth [37–39] and in protecting children from malnutrition symptoms [40] indicate that the microbiome could also play a pivotal role in protein homeostasis. However, the importance of commensals in protein homeostasis and in directing food choices has not been directly addressed.

In this study we show that yeast and AA preferences are driven by dietary deprivation from essential AAs (eAAs). While the absence of a single eAA is sufficient to induce a potent yeast appetite, removal of other important nutrients from the diet does not lead to an increase in yeast preference. The fly, however, is not specialized in detecting the identity of the missing AA. Flies rendered auxotrophic for a nonessential AA (neAA) display a strong yeast appetite upon deprivation of this artificially engineered eAA. Furthermore, we show that the presence of commensal bacteria abolishes the yeast appetite and the strong decrease in egg laying induced by the removal of eAAs. Commensal bacteria also have a strong phagostimulatory effect that is likely to aid the replenishment of gut bacteria. Using gnotobiotic animals, we show that the effect of commensals on yeast appetite is due to the concerted action of *Acetobacter pomorum* with *Lactobacilli*. Finally, we test the hypothesis that commensal bacteria alter feeding decisions by providing eAAs to the host. We find, however, no evidence that the decrease in eAA levels induced by dietary deprivation is ameliorated by the presence of commensal bacteria, suggesting that they may use a different mechanism to alter food choice. Our study identifies two key components driving food choice in *Drosophila*: eAAs and the gut bacteria species *Acetobacter pomorum* and *Lactobacilli*. Furthermore, we provide initial insights into their action on the host, highlighting the power of *Drosophila* for identifying key determinants underlying complex nutritional–microbial–behavioral interactions.

## Results

### Essential amino acids, but not other nutrients, control yeast and amino acid preference

**Fig 1:**
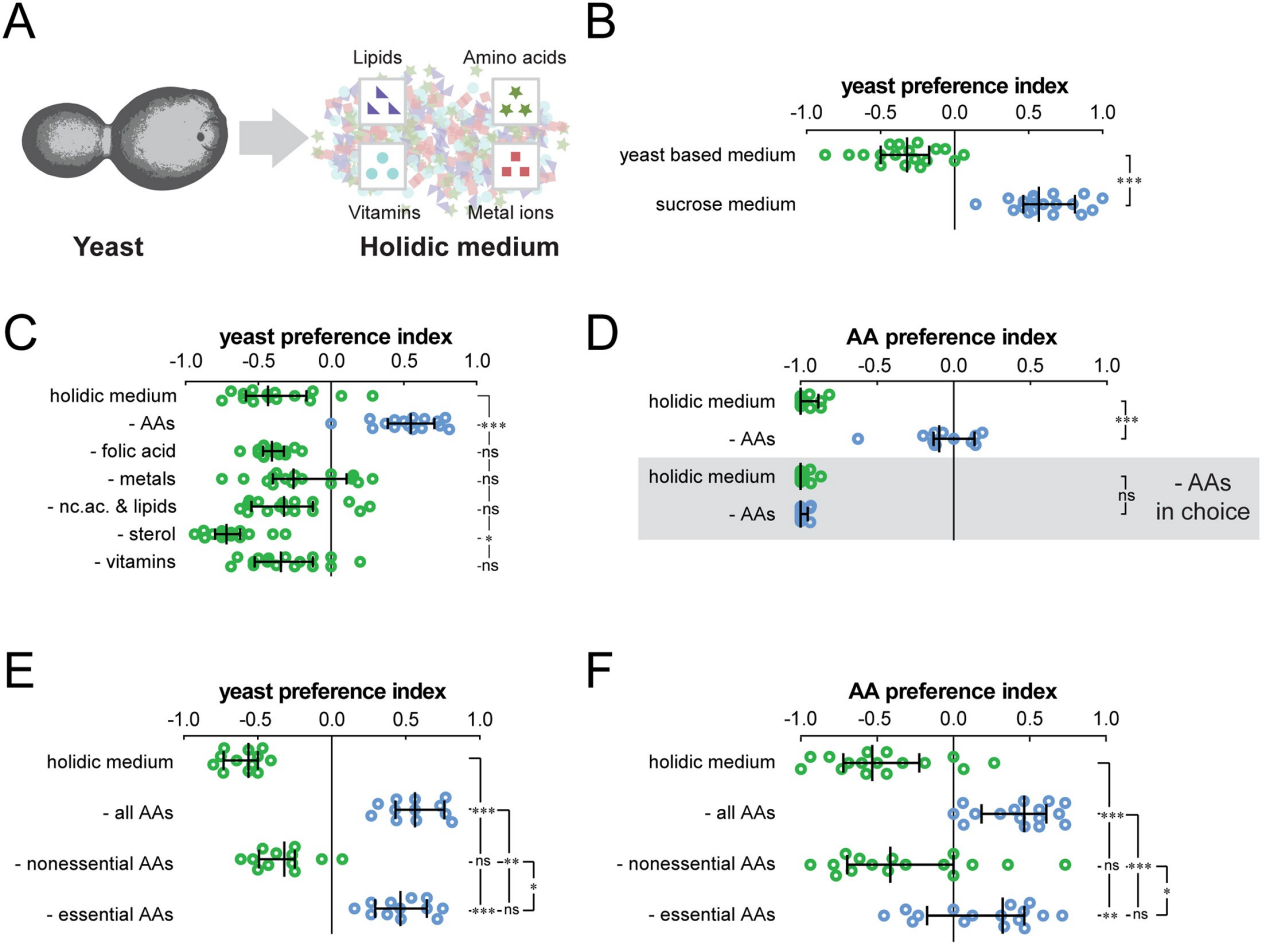
Flies specifically increase yeast and amino acid preference upon essential amino acid (eAA) deprivation. (A) The holidic diet allows the analysis of the impact of specific nutrients contained in yeast. (B) Yeast preference of flies kept on yeast-based medium and medium without yeast (sucrose medium). (C) Yeast preference of flies kept on holidic medium and holidic medium lacking different specific nutrients. (D) Amino acid (AA) preference of flies kept on full holidic medium and holidic medium lacking all AAs. (E and F) Yeast (E) and AA (F) preference of flies kept on complete holidic medium or holidic medium lacking all AAs, all nonessential amino acids (neAAs), or all eAAs. In (B), (C), and (E), flies were given the choice between sucrose and yeast. In (D) and (F), flies were either given the choice between holidic medium lacking AAs (sucrose option) or the holidic medium lacking sucrose (AAs option) and in (D) the sucrose option and holidic medium without sucrose and AAs (–AAs in choice). Circles represent yeast or AA preference in single assays, with a line representing the median and whiskers representing the interquartile range. *n* = 12–18. Significance was tested using the Kruskal–Wallis test followed by Dunn’s multiple comparison test. (B–F) Not significant (ns) *p* > 0.05, * *p* < 0.05, ** *p* < 0.01, *** *p* < 0.001. In this and the following Figs, green signifies diets with full eAA content and blue signifies diets lacking one or more eAAs. Underlying data used in this Fig are provided in S1 Data.

Yeast deprivation leads to a strong compensatory appetite for yeast [12] (Fig 1B). Given the complexity of this resource, it has not been possible to identify the nutrients that, when absent, trigger flies to ingest yeast. To answer this question, we decided to manipulate each nutrient present in yeast independently using a chemically defined (holidic) diet [7] (Fig 1A) and study their effects on feeding decisions using a two-color food choice assay [12,41]. The holidic medium is able to suppress yeast appetite to the same extent as a yeast-based medium (Fig 1B and 1C), supporting the idea that it provides the necessary nutrients to support adult behavior [7]. Removal of AAs from the holidic medium induced a potent yeast appetite, indistinguishable from that observed upon yeast deprivation (Fig 1B and 1C). Removing folic acid, metals, nucleic acids, lipids, sterols, or vitamins, however, did not lead to a significant increase in yeast appetite (Fig 1C). This effect stands in strong contrast to the clear effects on lifespan and egg production of removing these nutrients [7].

To identify the nutrients that flies select when deprived of AAs, we used the holidic diet in the choice paradigm. We gave the flies a choice between a holidic base diet containing sucrose without AAs and one containing AAs but no carbohydrates. AA deprivation shifted flies' preference from the sucrose-containing option towards the AA-containing option (Fig 1D). This shift in preference was specific to AAs since it was abolished by removal of AAs from the choice medium, whereas removing any other class of nutrients left the shift intact (Fig 1D and S1A Fig). These results suggest that similarly to yeast deprivation [42], upon AA deprivation, flies specifically select a diet containing AAs. In our paradigm, the decision to switch from eating sucrose to yeast or AAs is therefore guided by the absence of AAs, while the absence of other physiologically important dietary nutrients does not lead to an increase in yeast or AA appetite.

AAs can be broadly classified as either essential or nonessential. neAAs can be synthesized by the animal, allowing animals to be largely independent from dietary uptake of these important building blocks [43]. It is currently unclear whether animals sense these two types of AAs differently and if they have different effects on nutrient choice [44,45]. We tested this by manipulating AAs of each type independently. Removal of all eAAs from the diet induced a yeast (Fig 1E) and AA appetite (Fig 1F) that were indistinguishable from that observed upon removal of all AAs. The complete removal of neAAs, however, had no effect on nutrient choice (Fig 1E and 1F). Given that we adjust the total level of AAs to maintain a constant amount of nitrogen in the diet, these results also show that it is the identity of the AAs and not the nitrogen level in the diet that leads to changes in food choice. Intriguingly, AA deprivation induced a preference for both eAAs and neAAs, suggesting that the phagostimulatory power of AAs is not correlated with their nutritional importance, as indicated by previous studies [42] (S1B Fig). Taken together, these data strongly indicate that eAAs are specific mediators of protein and AA appetite and highlight the ability of animals to efficiently buffer the absence of neAAs.

### The absence of any single essential amino acid can induce a potent yeast appetite

**Fig 2:**
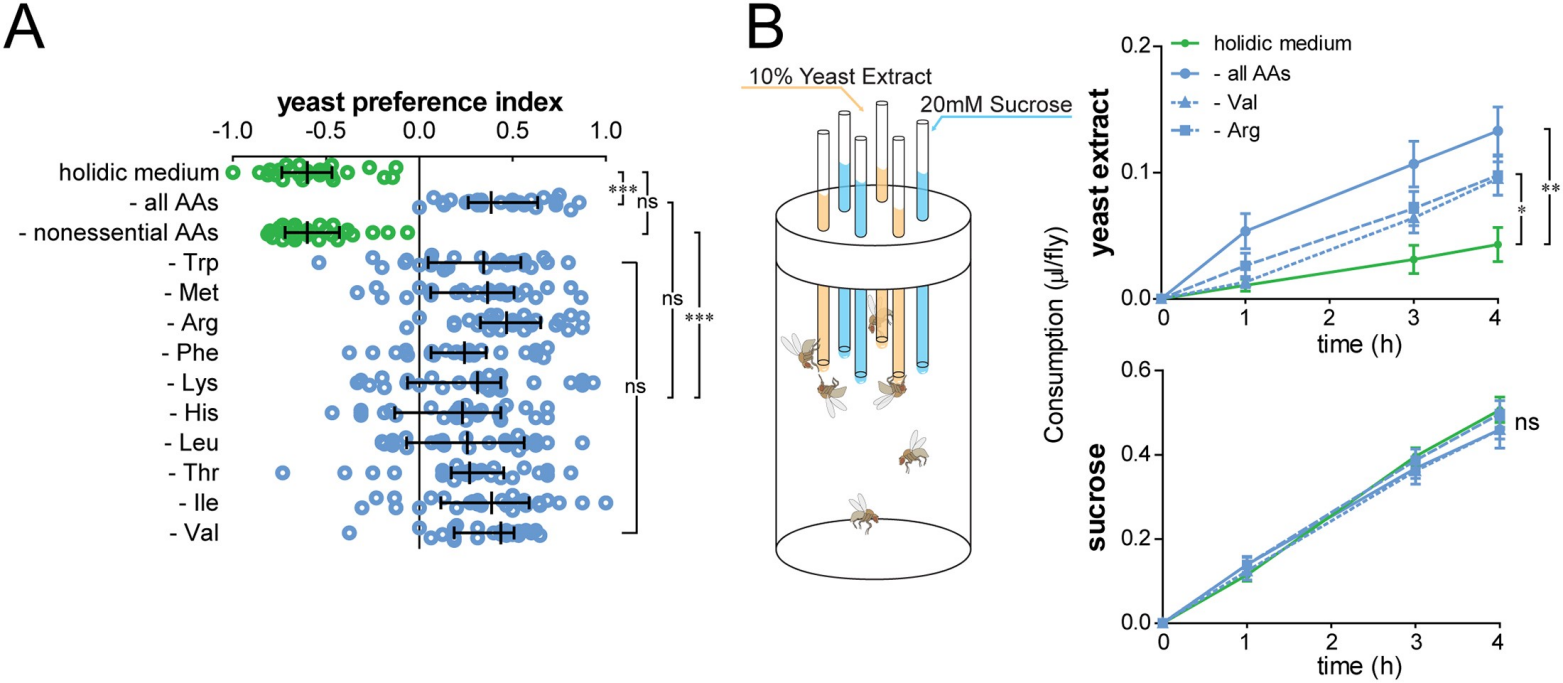
Flies specifically increase yeast appetite upon single essential amino acid (eAA) deprivation. (A) Feeding preference of flies kept on holidic medium or holidic medium lacking all amino acids (AAs), all nonessential amino acids (neAAs), or single eAAs in the context of no neAAs. Circles represent yeast preference in single assays, with a line representing the median and whiskers representing the interquartile range. *n* = 26. Significance was tested using the Kruskal–Wallis test followed by Dunn’s multiple comparison test. (B) Cumulative intake measurement of yeast extract and sucrose using the capillary feeder (CAFE) assay. Flies were prefed a holidic diet containing either all AAs, no AAs, all AAs except valine (Val), or all AAs except arginine (Arg). Dots represent means and error bars represent the standard error of the mean. *n* = 10. Significance was tested using the unpaired *t* test with Bonferroni correction for the intake volume at 4 h. For yeast extract intake in (B), Val and Arg deprivation have the same effect when compared to the complete holidic medium. There was also no significant effect of the different diets on sucrose intake. Not significant (ns) *p* > 0.05, * *p* < 0.05, ** *p* < 0.01, *** *p* < 0.001. Underlying data used in this Fig are provided in S1 Data.

Behavioral [45], physiological [9], and molecular studies [46] have suggested that different single AAs can vary widely in their potency to suppress protein appetite and to activate nutrient-sensitive pathways. We therefore took advantage of the unique possibility to manipulate single dietary AAs afforded by the holidic diet to remove every eAA individually from the diet and test the effect on food choice. Strikingly, removal of any eAA was sufficient to induce a clear increase in yeast choice (Fig 2A). The extent to which they did so did not differ, suggesting that each eAA has a similar impact on food choice. Furthermore, we quantified the effect of removing specific AAs from the diet on the intake of sucrose and yeast extract using a method to quantify food intake [47] (the capillary feeder [CAFE] assay; Fig 2B). Consistent with our results using the two-color assay, removal of either all AAs or single eAAs (arginine or valine) led to a specific increase in yeast extract intake without affecting carbohydrate intake (Fig 2B). In agreement with previous reports [16], these data indicate that the changes in food choice induced by AA deprivation in the two-color choice assay are due to an increase in yeast appetite and not to a decrease in sucrose intake. They further indicate that single eAAs are potent and specific nutritional modifiers of protein intake, highlighting their unique importance in controlling food choice.

### Loss of peripheral synthesis of neAAs increases yeast appetite

**Fig 3:**
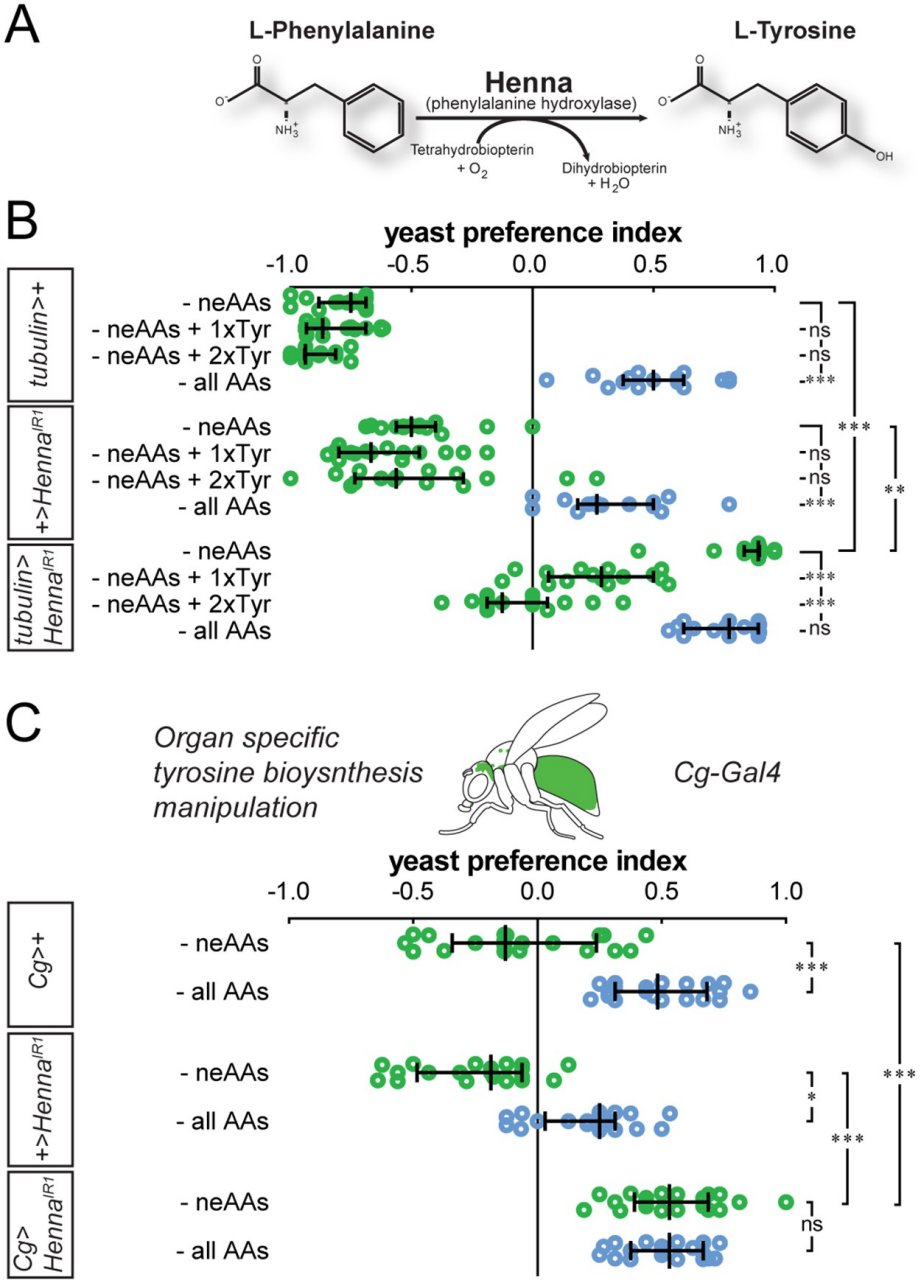
Loss of peripheral synthesis of nonessential amino acids (neAAs) increases yeast appetite. (A) Schematic depicting the biosynthesis of tyrosine (Tyr) in *Drosophila*. (B) Feeding preference of control and *Henna* whole-animal knockdown flies upon removal of all amino acids (AAs), all neAAs, or all neAAs with 1x and 2x Tyr added back. (C) Feeding preference of control and *Henna* fat body knockdown flies upon removal of either all neAAs or all AAs. (B and C) Circles represent yeast preference in single assays, with a line representing the median and whiskers representing the interquartile range. *n* = 15–20. Significance was tested using the Kruskal–Wallis test followed by Dunn’s multiple comparison test. Not significant (ns) *p* > 0.05, * *p* < 0.05, ** *p* < 0.01, *** *p* < 0.001. Underlying data used in this Fig are provided in S1 Data.

Animals can synthesize neAA in order to compensate for their absence from the diet. For example, tyrosine (Tyr) can be synthesized from phenylalanine (Phe) through the action of phenylalanine hydroxylase, which in *Drosophila* is encoded by the *Henna* gene (Fig 3A) [48]. In humans, mutations in phenylalanine hydroxylase cause phenylketonuria, the most common metabolic disease [49,50]. Patients with phenylketonuria suffer from elevated Phe and low Tyr titers, leading to severe complications including neurological and behavioral symptoms [51]. Strict adherence to a diet low in Phe and high in Tyr allows patients to lead an asymptomatic life, highlighting the impact of dietary AAs on human health [52].

We mimicked the genetic lesion leading to phenylketonuria by knocking down the *Henna* gene ubiquitously (S2A Fig), thus transforming Tyr from a neAA to an eAA. This allowed us to test if the capacity to homeostatically trigger changes in food choice is related to the specific identities of the ten eAAs or if it can be driven by low levels of any AA. While removal of dietary neAAs in control animals did not lead to the induction of a yeast appetite, the same dietary manipulation in *Henna* knockdown animals led to a strong yeast appetite (Fig 3B and S2B Fig). This increased yeast appetite was indistinguishable from that observed upon removal of all AAs. Supplementing the diet lacking neAAs with Tyr suppressed the preference of flies for yeast in a dose-dependent manner, indicating that the phenotype was specifically due to an acute lack of Tyr and not to other detrimental effects of our genetic manipulation (Fig 3B). Importantly, the addition of proline, a neAA which is not synthesized by phenylalanine hydroxylase, did not suppress the *Henna* phenotype, further emphasizing the specificity of the metabolic manipulation (S2B Fig). These results strongly suggest that flies can detect the absence of any limiting AA independent of their specific identity (eAA versus neAA).

In mammals, neAAs are mainly synthesized in the liver [53,54], and it is thought that in insects, the fat body fulfills a similar role [55–57]. We tested the importance of the fat body in guiding nutrient choice by interfering with the ability of this organ to synthesize Tyr. Knockdown of *Henna* using a fat body driver *Cg-Gal4* rendered the animal sensitive to the absence of dietary neAAs, with induction of a strong yeast appetite (Fig 3C). *Henna* knockdown in neurons or trachea, in contrast, did not change the behavioral sensitivity of flies to removal of all neAAs (S2C Fig), indicating that the effect observed with the fat body manipulation is tissue specific. However, *Cg-Gal4* has also been shown to drive expression in hemocytes [58]. It is thus possible that this cell type also contributes to Tyr synthesis and the observed behavioral phenotype. Taken together, these data further demonstrate that AAs, be they dietary or endogenously synthesized, are able to control yeast appetite. Furthermore, our data indicate that biosynthetically active organs are important regulators of food choice, suggesting that genetic metabolic conditions such as phenylketonuria could have effects on aspects of behavior such as nutrient-specific appetites.

### Commensal bacteria direct feeding decisions

**Fig 4:**
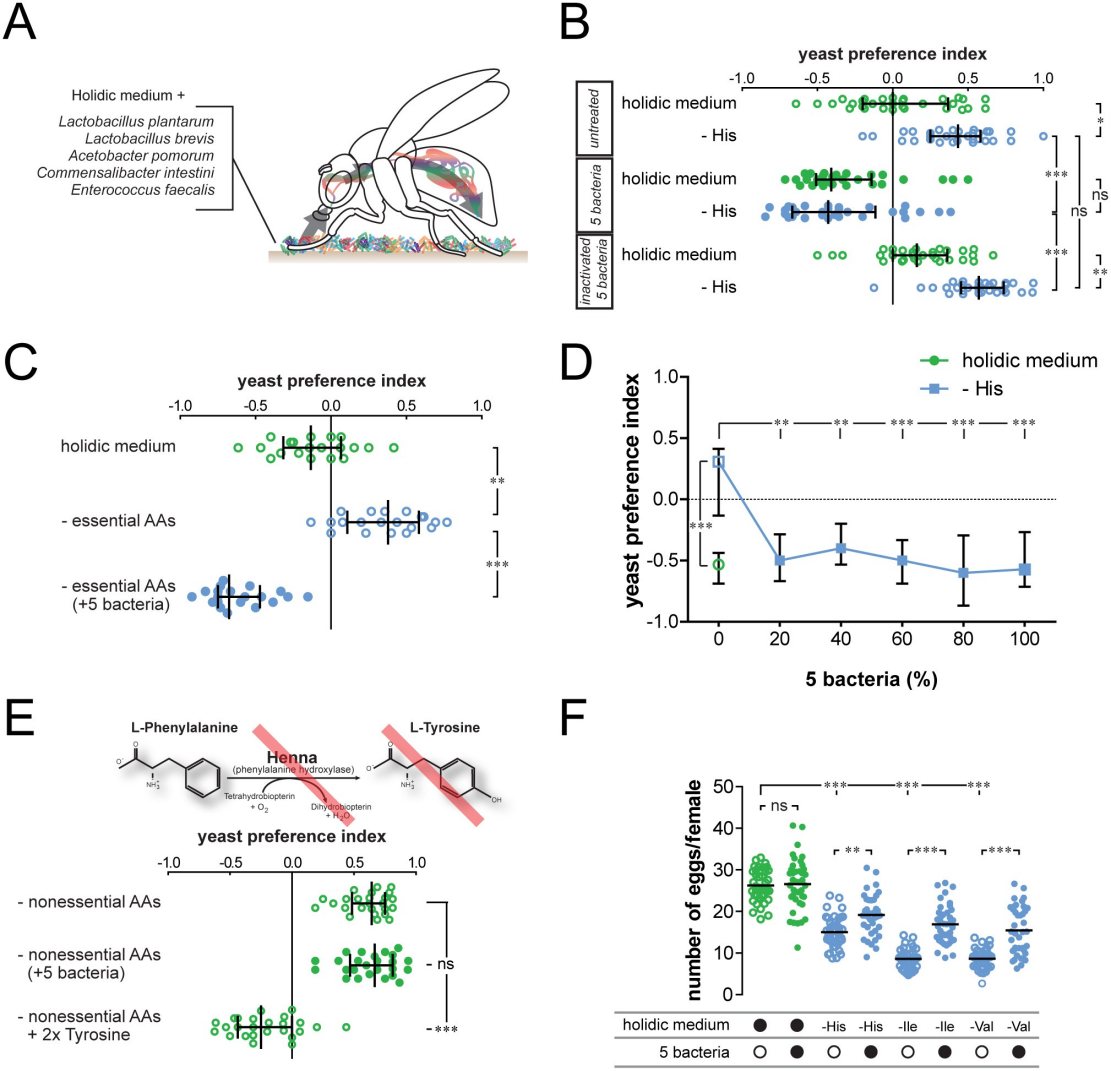
Commensal bacteria control food choice and egg laying. (A) Diagram depicting the strategy used to reconstitute the commensal population in the fly. (B) Yeast preference of animals kept on holidic diet with or without histidine (His). One group was not pretreated with the five commensals (untreated), the other was pretreated with the commensals (five bacteria), and the third was pretreated with inactivated commensals (inactivated five bacteria). (C) Yeast preference of animals kept on holidic diet, holidic diet without essential amino acids (eAAs), or holidic diet without eAAs pretreated with the commensal mix. (D) Yeast preference of animals kept on holidic diet with or without His and pretreated with varying concentrations of the commensal mix. (E) Yeast preference of *Henna* knockdown animals (*tubulin > Henna*^*IR1*^) kept on holidic diet lacking nonessential amino acids (neAAs), with or without pretreatment with the commensal mix, and holidic diet lacking neAAs with 2x tyrosine (Tyr) added back. (F) Number of eggs laid per female in 24 h of animals kept on holidic diet, holidic diet with all amino acids except His, isoleucine (Ile), or valine (Val), and with or without pretreatment with commensals. Black filled circles represent complete holidic medium or pretreatment with the bacteria mix. Open black circles represent flies that were not pretreated with bacteria mix. Amino acid (AA) deprivation is indicated as –His, –Ile or –Val. Data are pooled from two different rounds of experiments performed independently on different days. *n* = 39–40. (B, C, and E) Circles represent yeast preference in single assays, with a line representing the median and whiskers representing the interquartile range. Filled circles represent assays in which flies had been pretreated with commensals. *n* = 20–30. (D) Points represent median yeast preference and error bars represent the interquartile range. *n* = 15. (F) Circles represent eggs laid in single assays, with the line representing the mean. (B–E) Significance was tested using the Kruskal–Wallis test followed by Dunn’s multiple comparison test and in (F) using the one-way analysis of variance test followed by Bonferroni’s multiple comparison test (B–F) Not significant (ns) *p* > 0.05, * *p* < 0.05, ** *p* < 0.01, *** *p* < 0.001. Underlying data used in this Fig are provided in S1 Data.

Mounting evidence indicate that commensal bacteria are important determinants of how nutrients are utilized [59,60]. As such, they modulate a large set of nutrient-sensitive traits. However, whether commensals influence the selection of specific dietary nutrients is currently unknown. We therefore set out to test the effect of commensals on nutrient choice in *Drosophila*. Importantly, the flies used in our experiments had a very low baseline gut microbe load (S3 Fig). This is likely due to the use of sterile media and the fact that upon serial passage to new food, adult flies lose a large part of their microbiota [61]. To test the effect of the microbiota on behavioral protein homeostasis, we removed one eAA (histidine [His]) from the holidic diet to increase the flies' preference for yeast and examined if they would show alterations in food choice when treated with a controlled microbiota (Fig 4A) (pure culture of five *Drosophila* gut bacteria strains: *Lactobacillus plantarum*^WJL^ [62], *Lactobacillus brevis*^EW^ [62], *Acetobacter pomorum* [62], *Commensalibacter intestini*^*A911T*^ [62], and *Enterococcus faecalis* [63]). Strikingly, in contrast to control flies, bacteria-treated flies did not show an increased yeast appetite upon His removal (Fig 4B). The effect of commensals was not limited to His but suppressed yeast appetite upon removal of any of the ten tested eAAs (S4 Fig). The effect of commensals on food choice was so strong that the flies with a reconstituted microbiome were even buffered against the removal of all eAAs from the diet (Fig 4C).

To test if the bacteria were merely acting as food or if they needed to be metabolically active, we tested the effect of inactivated bacteria on food choice. Inactivation abolished the ability of the bacteria to alter feeding decisions, suggesting that their activity is essential to drive the change in behavior (Fig 4B). The behavioral effect was specific to commensal bacteria, as it was not observed when a non-commensal bacterium (*Escherichia coli*) was used in the experiments (S5A Fig). That bacteria do not simply act as food is further supported by the fact that even when diluted, they are able to lower the preference for yeast (Fig 4D). Furthermore, to show that the effect of the bacteria is not confined to flies fed on the synthetic diet, we pretreated flies with decreasing amounts of yeast to induce a yeast appetite. As expected, flies fed with decreasing amounts of yeast showed an increase in yeast preference (S5B Fig). Similarly to the effect on AA-deprived flies, bacteria pretreatment reduced the yeast appetite of yeast-deprived flies when compared to non-pretreated controls (S5B Fig). This indicates that the effect of commensal bacteria on food choice is generalizable to ecologically relevant AA sources. Commensals are therefore strong modifiers of food choice behavior by buffering the animal from the effect of dietary lack of eAAs.

### Commensal bacteria can only buffer the absence of essential amino acids

Given the strong effect of commensals on food choice, we set out to test if they would also be able to suppress the food choice phenotype in flies which are impaired in Tyr synthesis. Surprisingly, addition of the five bacteria to the diet was not able to suppress the yeast appetite induced by neAA deprivation in *Henna* knockdown flies, while Tyr supplementation was able to suppress this appetite (Fig 4E). The bacteria mix was still able to reduce the yeast appetite induced by His deprivation in *Henna* knockdown flies, indicating that the bacteria are effective in this genetic background (S6 Fig). This indicates that the microbiota exerts its effect specifically in the context of eAA but not neAA depletion. This experiment also strongly suggests that the commensal pretreatment does not alter food choice in an indirect way (e.g., by suppressing the ability of flies to choose yeast or only increasing their preference for sucrose) and that the effect of the bacteria is not due to them serving as food, as these effects should also lead to a decreased yeast preference index in these flies. Commensal bacteria are therefore strong modifiers of food choice specifically in the context of eAA deficiencies.

### Commensal bacteria increase egg laying upon essential amino acid deprivation

Stem cell proliferation and differentiation is limited by the availability of eAAs [64–66]. In *Drosophila* this is most evident in the context of egg production, in which depletion of eAAs strongly reduces egg laying [7,9]. We therefore tested if commensals would also be able to affect egg laying. As shown above, depriving animals of three different eAAs (His, Ile, or Val) led to the induction of yeast appetite, which was strongly suppressed in animals pretreated with commensals (S4 Fig). Removal of any of these three eAAs significantly decreased egg laying when compared to the complete holidic diet (Fig 4F). Flies treated with the five bacteria, however, laid a significantly higher number of eggs in the context of a diet without single eAAs when compared to the flies without bacterial pretreatment (Fig 4F). The microbiota is therefore not only able to buffer the effect of removing eAAs in the context of food choice but also in terms of physiological traits such as the reduction of egg laying triggered by removing one eAA. Given the importance of fecundity for the fitness of the animal, this effect therefore suggests that in the adult fly the host–bacteria interaction is mutualistic.

### Commensal bacteria specifically increase yeast appetite in the context of eAA depletion

**Fig 5:**
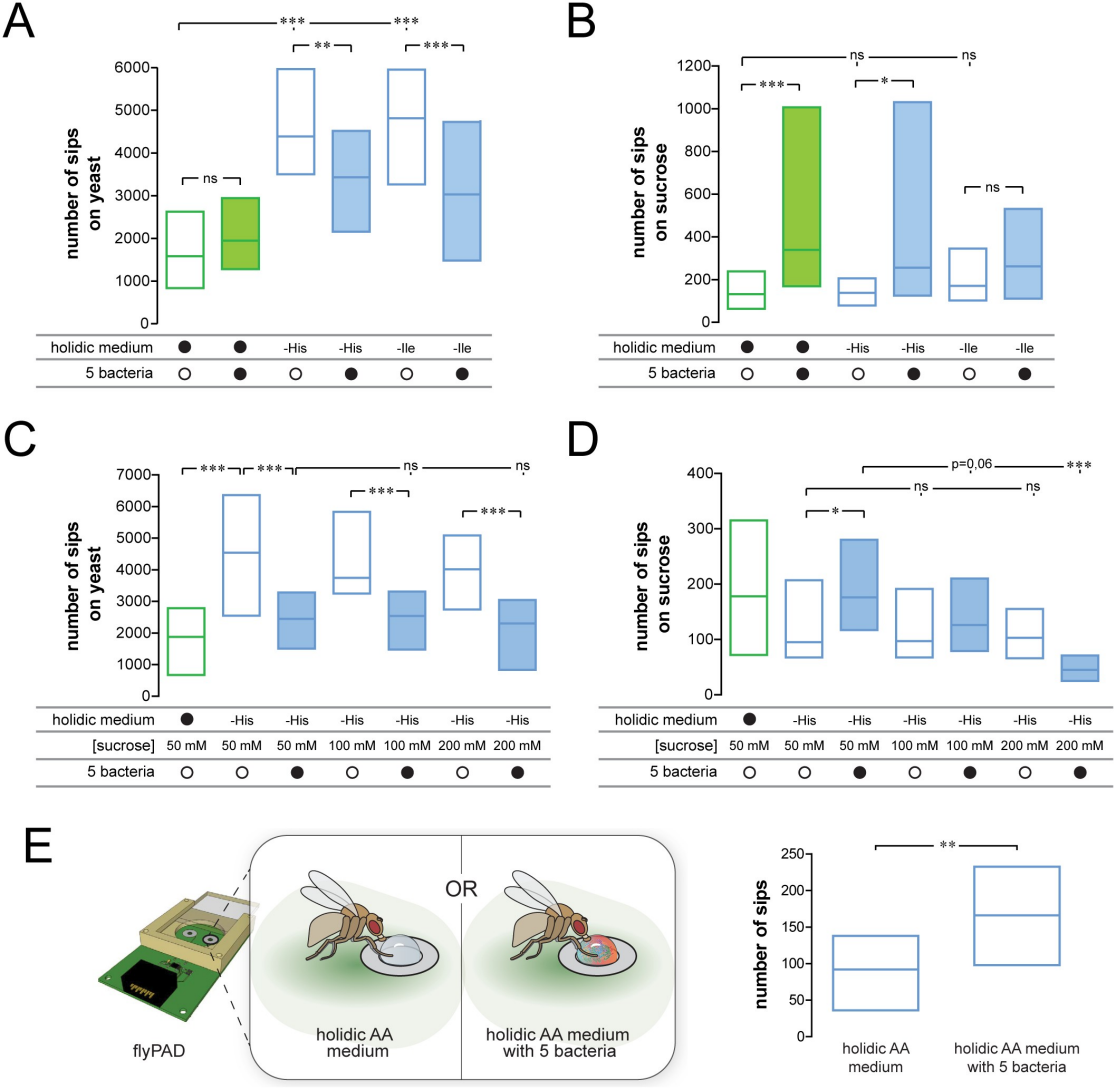
Commensal bacteria decrease yeast appetite upon essential amino acid (eAA) deprivation and are phagostimulators. Numbers of sips on yeast (A) or sucrose (B) of axenic flies prefed on holidic medium with different eAAs and with or without the commensal mix as measured using the flyPAD. *n* = 70–82. Numbers of sips on yeast (C) or sucrose (D) of axenic flies prefed on holidic medium containing different eAAs and different sucrose concentrations with or without the commensal mix as measured using the flyPAD. *n* = 25–37. (E) On the left, a diagram depicting the strategy used to test the phagostimulatory power of commensal bacteria. flyPAD drawing from [67]. On the right, numbers of sips of flies feeding from holidic base medium containing amino acids (AAs) with or without the commensal mix as measured using the flyPAD. *n* = 37–39. (A–E) Boxes represent upper and lower quartiles with the median. In this and other Figs, empty boxes represent non–bacteria-treated conditions and filled boxes represent bacteria-pretreated conditions. (A–D) Filled black circles represent a complete holidic medium or pretreatment with the bacteria mix. Open black circles represent flies not pretreated with bacteria mix. AA deprivation is indicated as –histidine (–His) or –isoleucine (–Ile) and in (C–D) sucrose concentration is indicated as values of 50 mM, 100 mM, or 200 mM. (A–C) Significance was tested using the Kruskal–Wallis test followed by Dunn’s multiple comparison test, in (D) using the Mann–Whitney test, and in (E) using the unpaired *t* test. Not significant (ns) *p* > 0.05, * *p* < 0.05, ** *p* < 0.01, *** *p* < 0.001. Underlying data used in this Fig are provided in S1 Data.

To separately analyze the effects of the microbiome on yeast and sugar feeding, we chose to use the flyPAD assay [67]. Furthermore, to fully control the microbial conditions of the experiments, we performed the flyPAD experiments starting with flies kept in an axenic state, which ensured that our microbiome reconstitutions resulted in gnotobiotic flies. Flies kept on a full holidic diet and pretreated with the five bacteria did not show an increase in yeast feeding when compared to the germ-free controls (Fig 5A), but they showed an increase in sucrose feeding (Fig 5B). The sucrose effect is reminiscent of previous reports that the gut microbiota can increase food intake in flies [60]. In agreement with the data generated using the CAFE assay, both His and Ile deprivation led to a specific increase in yeast feeding in the axenic flies (Fig 5A), while sucrose feeding was unaltered (Fig 5B). Compared with germ-free flies, the gnotobiotic flies pretreated with the five gut bacteria showed a highly significant decrease in yeast feeding, corroborating the hypothesis that the microbiota suppresses yeast appetite (Fig 5A).

In contrast to the effect on yeast appetite, the increase in sugar feeding observed in the flies treated with bacteria was more variable. While a significant effect was observed in the His-deprived flies, Ile-deprived flies did not show an increase in sucrose feeding (Fig 5B). These data support the idea that commensal bacteria specifically change food choice by decreasing yeast appetite in eAA-deprived flies.

It has been indicated that the microbiota competes with their host for the availability of sugars in the diet [36]. To test this hypothesis and rule out that the effect on yeast choice is due to the observed increase in sugar intake, we increased the amount of sugar in the holidic diet. Adding increasing amounts of sucrose to the diet decreased the sugar appetite, suggesting that the bacteria were indeed reducing the sugar available to the fly from the diet (Fig 5D). Importantly, neither the levels of sucrose in the diet nor the level of sugar feeding affected the yeast appetite, showing that these macronutrient appetites are independently regulated (Fig 5C and 5D). Therefore, while a decrease in the sugar content of the food could account for the previously reported increase in food intake caused by gut bacteria [60], this effect is not related to the changes in food choice we describe. Gut bacteria therefore use an independent mechanism to specifically reduce the yeast appetite of the host.

### Flies eat more vigorously from food containing commensal bacteria

Flies rely on the continuous replenishment of their microbiome through feeding [61]. If commensal bacteria provide protection against eAA depletion, one might expect flies to prefer ingesting food containing commensals. We therefore set out to compare the appetite of flies towards food with or without commensal bacteria using the flyPAD (Fig 5E). In agreement with our hypothesis, flies ate more vigorously from a food source containing the commensal bacteria when compared to the same food without commensals (Fig 5E). Flies are therefore able to increase feeding behavior when bacteria are present in the food. This suggests that flies are able to actively modulate their feeding behavior to replenish or modify their microbiota in order to profit from the physiological benefits of the commensals.

### *Acetobacter pomorum* acts together with *Lactobacilli* to modify food choice

**Fig 6:**
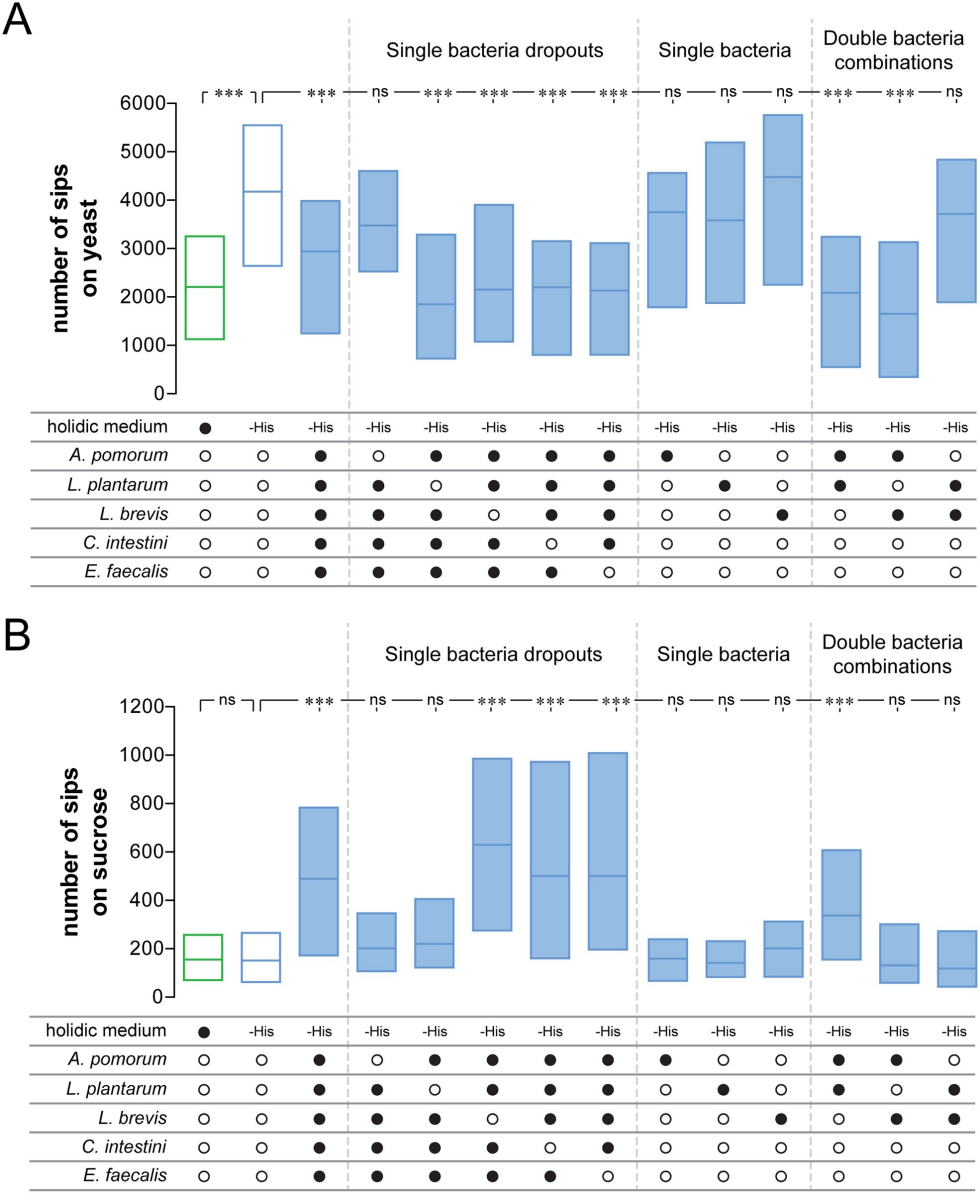
*A*. *pomorum* and *Lactobacilli* are sufficient to modify yeast appetite. Numbers of sips on yeast (A) or sucrose (B) as measured using the flyPAD of axenic flies prefed on complete holidic medium or holidic medium without histidine (His) and pretreated with different bacterial mixes. Filled black circles represent the complete holidic medium or presence of specific bacteria in the pretreatment mix. Open black circles represent the absence of specific bacteria in the pretreatment mix. His deprivation is indicated as –histidine (–His). Boxes represent upper and lower quartiles with the median. *n* = 46–120. Significance was tested using the Kruskal–Wallis test followed by Dunn’s multiple comparison test. Not significant (ns) *p* > 0.05, *** *p* < 0.001. Underlying data used in this Fig are provided in S1 Data.

Our data suggest that specific bacteria directly act on host physiology and behavior and provide evidence contrary to a generalized effect of bacterial material. We therefore decided to use the gnotobiotic model to identify which bacteria in the mix were producing the change in feeding behavior in eAA-deprived animals. To do so, we first removed each species separately from the mix and tested if the reduced sets could suppress the yeast appetite of His-deprived flies. While removal of *Acetobacter pomorum* (*Ap)* abolished the capacity of the mix to suppress yeast appetite, removal of any of the other four species had no effect (Fig 6A). *Ap* alone, however, is not sufficient to change yeast appetite, indicating that it acts in concert with other bacteria in the mix. Given that *Lactobacilli* act together with *Ap* to alter metabolite composition in flies [68], we decided to test if *Ap* together with *Lactobacillus plantarum* (*Lp)* or *Lactobacillus brevis (Lb)* are sufficient to alter yeast appetite. Indeed, the combination of *Ap* with either *Lp* or *Lb* is sufficient to suppress the yeast appetite induced by deprivation from either His or Ile (Fig 6A and S7 Fig). This result also explains why removing either *Lp* or *Lb* from the five-bacteria mix had no effect, as these species seem to act redundantly. Furthermore, neither *Lp*, *Lb*, nor the combination of both change feeding behavior, highlighting the specificity of the combined *Ap*–*Lactobacilli* effect on yeast appetite (Fig 6A).

The same approach allowed us to conclude that *Ap* and *Lp* act together to increase sugar appetite (Fig 6B). In contrast to the effect on yeast appetite, the *Ap–Lb* combination has no effect on carbohydrate consumption (Fig 6B). This reinforces the previous data showing that yeast appetite is independent of sugar appetite. Taken together, these data show that *Acetobacter pomorum* can act together with either *Lactobacillus plantarum* or to a certain extent with *Lactobacillus brevis* to change food selection.

### Commensal bacteria do not seem to change the levels of eAAs in the host

**Fig 7:**
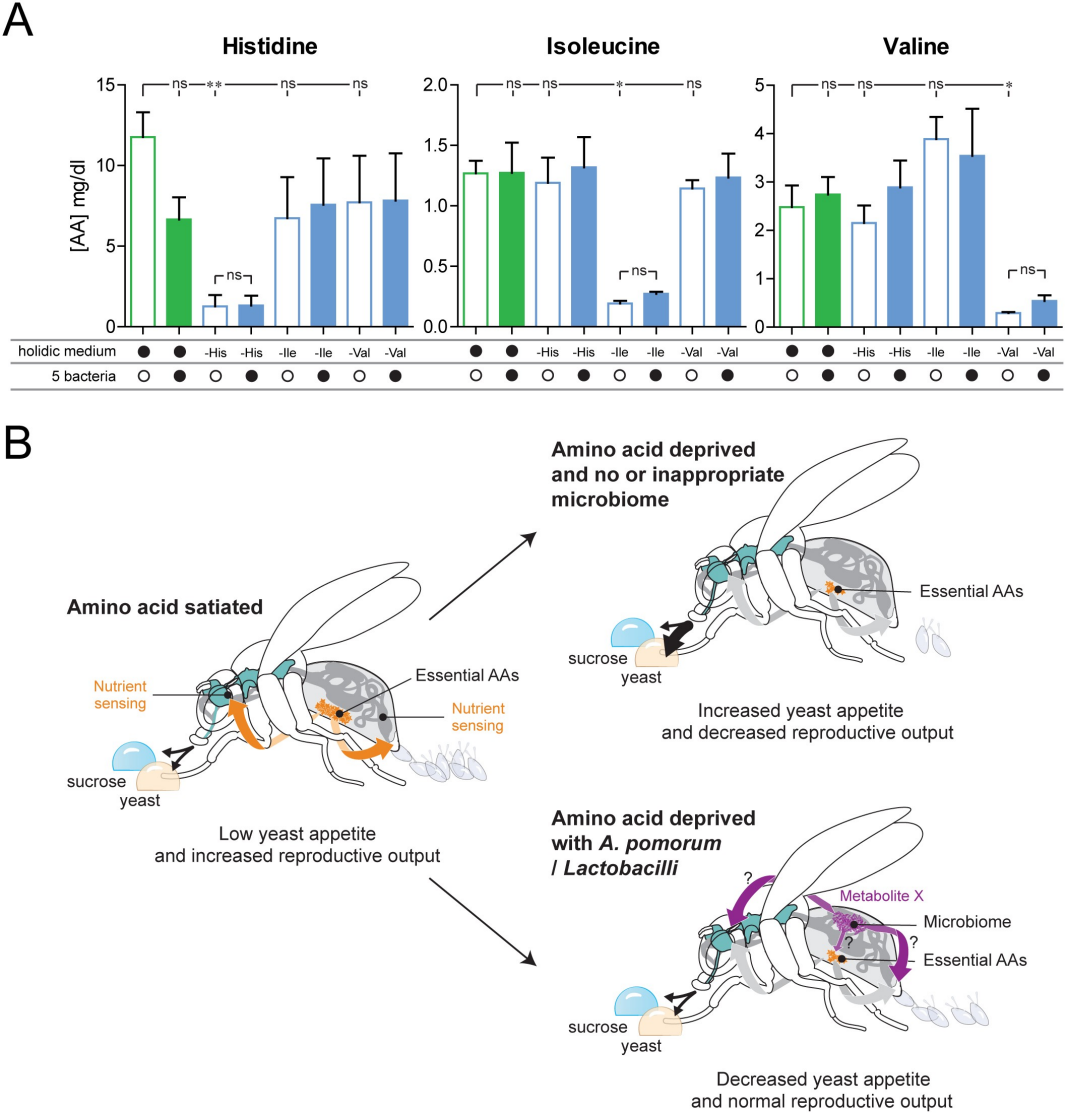
Commensal bacteria do not seem to change the levels of essential amino acids (eAAs) in the host. (A) Histidine (His), isoleucine (Ile), and valine (Val) concentrations in the heads of flies prefed on complete holidic medium (green) or holidic medium lacking His, Ile, or Val (blue), without (empty columns) or with (filled columns) commensals pretreatment. The columns represent the mean and the error bars the standard error of the mean of three independent experiments. Filled black circles represent complete holidic medium or pretreatment with the bacteria mix. Open black circles represent no pretreatment with bacteria mix. Amino acid (AA) deprivation is indicated as –histidine (–His), –isoleucine (–Ile), or –valine (–Val). Significance was tested using the unpaired *t* test with Bonferroni correction. Not significant (ns) *p* > 0.05, * *p* < 0.05, ** *p* < 0.01. (B) Model of the impact of eAAs on food choice and reproduction, depending on the presence of the microbiota of the host. The nervous system is highlighted in turquoise, AAs in orange, and commensal bacteria in purple. Arrow weight from the proboscis to the food drops indicate amount of feeding, and the number of eggs reflect the reproductive output. The orange and purple arrows indicate potential effects of eAAs and metabolites, respectively, at the level of the nervous and reproductive systems. Metabolite X refers to a hypothetical metabolite mimicking the presence of AAs. Underlying data used in this Fig are provided in S1 Data.

The ability of the commensal bacteria to compensate for the effect of eAA deprivation on yeast appetite and egg laying suggests that the bacteria could supply the host with eAAs, thus buffering the animal from the absence of these important nutrients in the diet. Such an effect would be reminiscent of the role of the *Buchnera* endosymbiont in aphids, which allows this insect to thrive while feeding on sap, which contains very low amounts of AAs [69]. We tested this hypothesis by depriving flies from three different eAAs (His, Ile, and Val) and comparing the levels of free AAs in the heads of flies that had been either pretreated or not with the five-bacteria mix. We decided to focus on the AA levels in heads to avoid effects due to changes in the number of eggs carried by the fly and because of evidence that nutrient sensing could act at the level of the brain of the fly to change food preference [12]. His, Ile, or Val deprivation lead to a drastic decrease in the levels of these three AAs in head extracts (Fig 7A), which is likely to cause the previously observed increases in yeast appetite (Fig 2). This effect was specific to the manipulated AAs, as the levels of nonmanipulated AAs neither increased nor decreased (Fig 7A). AA-satiated flies treated with the bacterial mix did not show an increase in His, Ile, or Val. Surprisingly, deprived flies continued having very low titers of the measured eAA independent of the bacterial pretreatment (Fig 7A). This stands in contrast to the clear effect of the bacterial pretreatment on yeast preference and egg laying (Fig 4 and S4 Fig). Our failure to observe changes in eAA levels induced by bacterial pretreatment opens the intriguing possibility that the commensal bacteria modify food choice and egg laying through an AA-independent mechanism (Fig 7B).

## Discussion

The complexity of natural foods makes it very difficult to identify the nutrients which guide behavior and physiology. Furthermore, while the microbiota has been suggested to affect behavior, until now its influence on specific nutrient appetites has not been explored. The present study identifies two factors as strong and specific modulators of feeding decisions and reproduction: eAAs and commensal bacteria (Fig 7B).

### Amino acids as potent modulators of protein appetite

Multiple nutrients, including AAs, metals, vitamins, and sterols have been shown to be nutritional modulators of life history traits [7]. Given that in an ecological setting, yeast is likely to be an important nutritional source of these nutrients, it is therefore surprising that the animal only develops a yeast appetite upon the restriction of AAs. One reason might be that animals have not evolved strategies to regulate the intake of all nutrients separately but just specific ones. This could be explained by the fact that in an ecological setting, animals do not need to react to the lack of each nutrient independently as these distinct nutrients are found together in nature in the form of food. If yeast is the ecologically relevant source of most nutrients required for the fly, then the animal could use the lack of internal AAs as a proxy for the concomitant lack of other nutrients, such as minerals, metals, and vitamins. The increase in yeast appetite triggered by AA deprivation would thus be sufficient to compensate for the lack of other nutrients. This would highlight that while synthetic diets are invaluable tools for studying the impact of nutrients on physiology and behavior, the results obtained from such studies always need to be interpreted in the context of ecologically relevant food sources such as yeast. It is also possible, however, that foods other than yeast can serve as sources for specific nutrients. Further focused analyses of the effect of specific nutrient classes on behavior will be required to identify the full capacity of *Drosophila* to maintain nutrient homeostasis.

### Signaling mechanisms and neuronal circuits underlying amino acid sensing

How do flies sense the internal deficiency of AAs and what are the circuit mechanisms allowing them to increase yeast or AA intake upon this nutritional restriction? The brain should be able to detect changes in AA concentrations given that the concentration of free eAAs in the head drops dramatically upon their removal from the diet. Changes in behavior could therefore either be informed by direct sensing of internal AA titers by the nervous system and/or by detecting a signal released by peripheral tissues as response to lack of AAs (Fig 7B). Two molecular mechanisms have been proposed as mediating neuronal AA sensing: the TOR and GCN2 pathways. In *Drosophila*, neuronal TOR signaling has been proposed to influence food selection [12], and in *Drosophila* and vertebrates, GCN2 has been proposed to direct behavioral nutrient homeostasis by mediating post-ingestive neuronal sensing of AAs [70–72]. The involvement of neuronal GCN2 in nutrient selection in vertebrates has, however, been recently challenged [73]. We therefore have only very rudimentary clues as to what could be the mechanisms mediating internal sensing of AA availability and subsequent changes in behavior. Our data that flies can behaviorally react to the absence of a genetically engineered neAA deficiency suggest that whatever the sensing mechanism, it has to be able to sense the absence of any AA. Intriguingly, at the molecular level, nutrient-sensing pathways such as the TOR pathway have been proposed to mainly react to specific AAs [46]. Our data suggest that nutrient-sensing pathways could have a much broader spectrum of action. Alternatively, the sensing of AA deficiencies could rely on different molecular mechanisms, which could for example detect the decrease in translation induced by the lack of AAs. Such a decrease could either be sensed per se or could lead to a decrease (or increase) of specific translation products, which could serve as signals to alter behavior. Pinpointing the site and cellular substrate of AA sensing as well as the underlying molecular mechanisms remains a key challenge in the field of nutrient homeostasis.

At the circuit level, the lack of AAs is likely to lead to a change in chemosensory processing that would lead to a change in nutrient preference. For proteins, such changes have been proposed in locusts [74]. In *Drosophila*, chemosensory neurons have been shown to be directly modulated by the internal energy state of the animal [13]. Furthermore, mating has been shown to modulate salt taste processing using the same circuit as yeast intake [14]. How internal AA states affect yeast and AA chemosensory processing, however, still remains to be elucidated. The main obstacle is the lack of information on the identity of the chemosensory neurons mediating yeast and AA feeding in *Drosophila*. Identifying these neurons and analyzing how yeast perception is modulated by internal state should allow us to better understand how the internal AA state directs feeding decisions.

### Host–microbiota interactions in nutrient choice

The extent to which the microbiota affects specific nutrient appetites has not been previously explored. We show that when flies are AA challenged, commensal bacteria reduce their compensatory yeast appetite. The increase in sugar appetite observed in flies harboring commensals further decreases the ratio of protein to carbohydrate intake, an important determinant of life history traits in animals, including vertebrates [2]. Given that a reduction in yeast and AA intake leads to an increase in lifespan, our observation that commensal bacteria reduce the intake ratio of proteins to carbohydrates could account for the shorter lifespan of axenic flies [75]. It is interesting to note that the flies harboring commensals are able to increase their reproductive output (our study and [76]) despite their lower protein intake. Commensal bacteria could therefore have a highly beneficial impact on the fly, enabling it to simultaneously maximize lifespan and reproductive output.

The increase in sugar appetite observed in gnotobiotic flies can be simply explained by the bacteria utilizing the sugar in the food and therefore inducing a carbohydrate deficit in flies. The decrease in yeast appetite, however, is more difficult to explain. One simple possibility could have been that these bacteria act as nutrients, which has been proposed for yeast and other fungi [77]. Our findings that inactivated bacteria do not induce a change in feeding behavior, that bacteria are not able to suppress the yeast appetite induced by neAAs when we perturb the function of *Henna*, and that only specific commensal bacteria change yeast appetite, all indicate that the microbiota acts in a very specific way to alter food choice. Furthermore, previous data that microbes can improve the uptake of AAs [77,78] are not sufficient to explain the suppression of yeast appetite in flies pretreated with commensals, as we use holidic media completely devoid of eAAs. A key question is thus: what could be the mechanisms by which gut bacteria change yeast appetite and increase egg laying?

### Mechanisms by which gut bacteria could control nutrient homeostasis in the host

Intracellular symbiotic bacteria are known to provide eAAs in other insects [69], and gut bacteria have been shown to provide significant amounts of eAAs in vertebrates, including humans [79]. A straightforward hypothesis would therefore be that these two bacteria are able to provide the fly with eAAs. However, we were not able to detect changes in the levels of free eAAs in flies pretreated with the commensal mix. In spite of this, we are reluctant to completely rule out that the microbiota acts on yeast appetite and egg laying by providing eAAs. It is possible, for example, that in an eAA-deprived situation, bacterially produced eAAs are immediately utilized without increasing the pool of free AAs. In such a model, bacterially derived eAAs would be fully allocated to sustain reproduction as well as alleviate the process which triggers changes in yeast appetite upon eAA deprivation. In such a situation, it is conceivable that one would not be able to measure an increase in free eAAs provided by the bacteria. Our data, however, suggest that commensal bacteria do not act by providing eAAs to the host. What could be alternative mechanisms by which they influence behavior and egg production? They could secrete metabolites that help the host to increase its ability to use its remaining AAs, thereby buffering the fly from the effects of dietary eAAs. Intriguingly, both yeast appetite and reproduction are thought to be regulated by the nutrient-sensitive TOR pathway [12,80–83], and commensals have been shown to be able to modulate this pathway [37]. It is therefore possible that these bacteria act directly on nutrient sensing pathways by releasing metabolites that mimic the availability of eAAs (Metabolite X in Fig 7B). Distinguishing between these hypotheses will require comprehensive metabolome analyses of flies in different bacterial and nutrient states as well as careful genetic and behavioral studies, both at the level of the host and the bacteria.

The metabolic repertoire of an organism is evolutionarily fixed in its genome. As such, it represents a static set which can mainly be modulated by transcriptional control. The observation that flies ingest more food containing commensal bacteria suggests that they might be able to direct their feeding behavior to replenish or maintain a specific microbiome composition. It is therefore attractive to speculate that the dynamic nature of the microbiome in flies paired with the ability to modulate the replenishment of gut microbes through feeding could allow them to extend and adapt their metabolic repertoire by exploiting that of the microbiome [84]. This ability could partially explain the success of *Drosophila* in adapting to a wide range of habitats.

Our understanding of how the microbiota influences behavior remains extremely rudimentary. In vertebrates, this task is made especially daunting by the complexity of their microbiota. *Drosophila*, on the other hand, has proven to be an especially powerful model for understanding microbe–host interaction because of the ability to isolate a single bacterial species promoting physiological effects such as improved growth [85,86]. Especially in vertebrates, many effects of the microbiome on the host, however, are likely to rely on interactions among different microbial species. Our finding that *Ap* acts together with *Lactobacilli* to influence food choice provides a powerful system for not only understanding how microbes act on the host to influence brain function but also how microbes cooperate to shape complex host traits. Microbes could act together by exchanging metabolites to act on the host. Alternatively, one bacterium could support the growth and survival of the other in nutritionally challenging situations, allowing it to exert its behavioral effect. The identification of these two bacterial species as mediators of food choice paired with the powerful genetic toolkit available in *Drosophila* provides a unique opportunity to identify the mechanisms by which microbes interact to shape the behavior of the host.

### The importance of nutritional–microbial interactions in influencing host behavior across phyla

Our findings highlight a new function of the microbiota in modulating nutrient-specific appetites. Given that in *Drosophila*, AA state not only controls food intake but also more complex behavioral features, such as risk taking [16], the microbiota could influence behavior beyond feeding. Furthermore, because AAs and nutrient sensing play a pivotal role in controlling physiology, neurodevelopmental disorders [87,88], and behavior across metazoans, such mechanisms could be conserved across phyla. Nutrition could therefore provide a framework for understanding how the microbiome influences behavior, disease, and physiology across phyla. Our findings highlight the power of the *Drosophila* model for dissecting complex nutritional–microbial–behavioral interactions and suggest the intriguing possibility that commensal bacteria influence behavior and brain function in invertebrates and vertebrates by tapping into the nutrient-sensing abilities of the nervous system.

## Materials and methods

Methods and protocols for Drosophila rearing, media preparations, and microbial manipulations are available as a collection in protocols.io dx.doi.org/10.17504/protocols.io.hdtb26n.

### *Drosophila* stocks and genetics

Unless stated otherwise, all experiments were performed with mated *w*^*1118*^ female flies. Ubiquitous (*tubulin-Gal4* [89]), pan-neuronal (*elav-Gal4* [90]), tracheal (*btl-Gal4* [91]), or fat body (*Cg-Gal4* [92], BL #7011) expression of RNAi delivering transgenes against *Henna* (*CG7399*) was achieved by crossing Gal4-carrying female flies with three independent UAS-*Henna*-RNAi stocks (*Henna*^*IR1*^: VDRC #35240; *Henna*^*IR2*^: NIG-RNAi #7399R-3; *Henna*^*IR3*^: BL #29540). The full genotypes of experimental flies are listed in S1 Table.

### *Drosophila* rearing, media, and dietary treatments

Flies were reared on yeast-based medium (YBM) (per liter of water: 8 g agar [NZYTech, PT], 80 g barley malt syrup [Próvida, PT], 22 g sugar beet syrup [Grafschafter, DE], 80 g corn flour [Próvida, PT], 10 g soya flour [A. Centazi, PT], 18 g instant yeast [Saf-instant, Lesaffre], 8 ml propionic acid [Argos], and 12 ml nipagin [Tegospet, Dutscher, UK] [15% in 96% ethanol] supplemented with instant yeast granules on the surface [Saf-instant, Lesaffre]). To ensure a homogenous density of offspring among experiments, fly cultures were always set with 5 females and 4 males per vial and left to lay eggs for 7 d. Flies were reared in YBM until adulthood. Holidic media (HM) were prepared as described previously [7] using the HUNTaa formulation without food preservatives, with the exception of the HM used for pretreating axenic and gnotobiotic flies, for which we used an HM with an improved AA composition [93]. The different HM used in this study are described in S2 Table and S3 Table. In all experiments where we refer to all neAAs removal, L-glutamate was still present in the diet in order to prevent any possible adverse effects in neuronal function. Sucrose medium consisted of Kleenex tissue soaked with 5 ml of a 100 mM sucrose (Sigma-Aldrich, #84097) solution. For all experiments using the HM, the following dietary treatment protocol was used in order to ensure a well-fed state and minimize the microbial load in the flies [61] (S8 Fig): groups of 1–5-d-old flies (16 females and 5 males) were collected into fresh YBM-filled vials and transferred to fresh YBM after 48 h. Following a period of 24 h, flies were transferred to different HM for 72 h and immediately tested in the indicated assay. Flies treated using this protocol had a low titer of commensal bacteria (S3 Fig). For yeast dilution experiments presented in S5 Fig, flies were kept for 72 h prior to the behavioral assay on media containing 200 mM sucrose (Sigma-Aldrich, #84097), 2% agar (Difco, # 214530), and variable instant yeast concentrations: 5%, 2.5%, 1%, and 0% (Saf-instant, Lesaffre). After preparation, all yeast-based media were autoclaved before pouring into culture vials. Fly rearing, maintenance, and behavioral testing were performed at 25°C in climate-controlled chambers at 70% relative humidity in a 12-h light–dark cycle (Aralab, FitoClima 60000EH). Polypropylene fly vials (VWR, #734–2261) were used.

These protocols are available in the following collection in protocols.io dx.doi.org/10.17504/protocols.io.hdtb26n.

### Axenic *Drosophila* generation and rearing

The protocol to generate axenic *w*^*1118*^ fly cultures by sterilizing embryos was adapted from [94]: embryos were put for 2 min in 2.5% active chlorine (50% bleach) followed by 2 min in 70% ethanol and 2 min in autoclaved distilled water. The embryos were then transferred onto sterile food (autoclaved before pouring into culture vials) containing antibiotics (final concentrations: 416.7 μg/ml tetracycline [high dose], 41.67 μg/ml chloramphenicol, 41.67 μg/ml ampicillin, and 8.333 μg/ml erythromycin). In order to compensate for the developmental delay observed in axenic larvae [37], the yeast content of the medium was increased to 41.67 g per liter. Axenic *w*^*1118*^ flies were regularly transferred into vials containing freshly prepared, antibiotic-supplemented, high-yeast food (S8 Fig). The absence of bacteria was assessed by grinding flies in sterile 1x PBS and spreading the suspension on LB, MRS, or Mannitol plates. LB and MRS plates were incubated at 37°C and Mannitol plates at 30°C, respectively, before assessing the presence of bacterial colonies. The antibiotic treatment did not lead to any apparent malaise in the treated flies. Furthermore, to ensure that the antibiotics exposure would not directly affect the experimental animals, these were raised in sterile food without antibiotics (S8 Fig). Importantly, the results obtained using the gnotobiotic flies fully recapitulate the results obtained with the conventionally reared “low bacteria titer” flies.

All experiments in Figs 5A–5D, 6 and S7 were performed using axenic or gnotobiotic flies.

These protocols are available in the following collection in protocols.io dx.doi.org/10.17504/protocols.io.hdtb26n.

### Bacterial species and cultures

The following bacterial species and strains (kindly provided by François Leulier, IGFL, France, and Won-Jae Lee, SNU, South Korea) were used in this study: *Lactobacillus plantarum*^WJL^ [62], *Lactobacillus brevis*^EW^ [62], *Acetobacter pomorum* [62], *Commensalibacter intestini*^*A911T*^ [62], and *Enterococcus faecalis* [63]. *Lactobacilli* were cultured in 10 ml of liquid MRS medium (Sigma-Aldrich, #69966) in 14 ml culture tubes (Thermo Fisher Scientific, #150268) at 37°C for 24 h without agitation. *C*. *intestini*^*A911T*^ and *A*. *pomorum* were cultured in a liquid mannitol medium (3 g/l Bacto peptone [Difco, #0118–17], 5 g/l yeast extract [Difco, #212750], 25 g/l D-mannitol [Sigma-Aldrich, #M1902]) at 30°C for 48 h under 170 rpm agitation. *C*. *intestini*^*A911T*^ was cultured in 20 ml of medium in 50-ml tubes (Falcon), and *A*. *pomorum* was cultured in 200 ml of medium in 500-ml flasks. *E*. *faecalis* was cultured in 200 ml of liquid LB medium (Sigma-Aldrich, #L3022) in 500-ml flasks at 37°C for 24 h under 220 rpm agitation. Liquid cultures were set with colonies grown in fresh solid media (15 g/l agar [Difco, # 214530]).

These protocols are available in the following collection in protocols.io dx.doi.org/10.17504/protocols.io.hdtb26n.

### Inoculation of HM with bacteria

Prior to transferring the flies, each HM vial was inoculated with either single or different combinations of the following bacterial species: *L*. *plantarum*^*WJL*^ (6.4 x 10^4^ CFU), *L*. *brevis*^*EW*^ (5.31 x 10^3^ CFU), *C*. *intestini*^*A911T*^ (9.04 x 10^4^ CFU), *A*. *pomorum* (9.5 x 10^4^ CFU), *E*. *faecalis* (1.11 x 10^5^ CFU), and *E*. *coli* (0.924 x 10^9^ CFU). To prepare this mixture, the necessary volume of liquid culture for each bacterial species was centrifuged three times at 3,000 rpm for 10 min and repeatedly resuspended in 1x PBS. To exclude an effect from residual components of bacterial media, the equivalent volume of sterile bacterial media was centrifuged in parallel and used as a control. After the final centrifugation, both the control and the bacterial pellet were resuspended in sufficient 1x PBS to achieve an inoculation volume of 50 μl per vial. For the experiments with heat-inactivated bacteria, the bacterial suspension was incubated at 100°C for 10 min before inoculation in HM vials. The final suspensions were added to the surface of the HM and allowed to dry for approximately 1 h before the addition of flies. Note that even when not pretreated to be axenic, because of rearing protocol flies used in all experiments had a very low starting titer of internal microbes prior to inoculation (S3 Fig).

These protocols are available in the following collection in protocols.io dx.doi.org/10.17504/protocols.io.hdtb26n.

### Calculation of internal bacterial load of flies

Flies were surface sterilized to remove any bacteria that could be found on the cuticle by washing them in 70% ethanol followed by two washes in sterile 1x PBS. Flies were grinded in 1x PBS (500 μl/18 flies) and diluted 180X. The suspension was then plated on LB, MRS, or Mannitol medium. LB and MRS plates were incubated at 37°C and mannitol plates at 30°C before counting the number of bacterial colonies.

These protocols are available in the following collection in protocols.io dx.doi.org/10.17504/protocols.io.hdtb26n.

### Two-color food choice assay

Two-color feeding preference assays were performed as previously described [12]. Groups of 16 female and 5 male flies were briefly anesthetized using light CO_2_ exposure and introduced into tight-fit-lid Petri dishes (Falcon, #351006). For the yeast choice assays, the flies were given the choice between nine spots of 10 μl sucrose solution mixed with red colorant (20 mM sucrose [Sigma-Aldrich, #84097]; 7.5 mg/ml agarose [Invitrogen, #16500]; 5 mg/ml Erythrosin B [Sigma-Aldrich, #198269]; 10% PBS) and nine spots of 10 μl yeast solution mixed with blue colorant (10% yeast [Saf-instant, Lesaffre]; 7.5 mg/ml agarose; 0.25 mg/ml Indigo carmine [Sigma-Aldrich, #131164]; 10% PBS) for 2 h. For the defined nutrient-choice assays, flies were given the choice between HM lacking AAs and containing 20 mM sucrose mixed with red colorant (option 1: sucrose) and HM lacking sucrose and containing the nutrients required for the experiment mixed with the blue colorant (option 2). In these experiments, the agar concentration in the HM was changed to 1.5%. After visual inspection of the abdomen under the stereo microscope (Zeiss, Stereo Discovery.V8), each female fly was scored as having eaten either sucrose (red abdomen), yeast (blue abdomen), or both (red and blue or purple abdomen) media. The yeast preference index (YPI) for the whole female population in the assay was calculated as follows: (n_blue yeast_−n_red sucrose_) / (n_red sucrose_ + n_blue yeast_ + n_both_). Initially, dye-swap (red yeast versus blue sucrose choice) experiments were performed in parallel, and because the change of feeding preference was observed in both conditions, we opted to exclusively perform red sucrose versus blue yeast choice experiments. In all experiments, the observer was blind for both diet and genotype. All assays were performed between ZT6 and ZT9.

### CAFE assay

CAFE assays were based on a protocol previously described [14,47] with some adaptations. On the assay day, flies were anesthetized with CO_2_, sorted under a stereo microscope in groups of 18 females, and allowed to recover for 3 h at 25°C. The CAFE chamber consisted of a large plastic vial (50 x 100 mm) (Semadeni AG, #6128) with 6 5-μl glass capillaries (Hirschmann, #9600105) inserted through a foam lid. Capillaries were filled with 20 mM sucrose or 10% yeast extract (Sigma-Aldrich #1625) solutions and placed in an alternating circular fashion. Each group of flies was aspirated into a CAFE chamber, and during the 4 h of the assay, four experimental readings per capillary were scored (t_0_, t_0+1 h_, t_0+3 h_, and t_0+4 h_) to determine consumption. In order to correct for evaporation, each set of experimental chambers was accompanied by an empty chamber (no flies). Total sucrose or yeast extract consumption per time point was determined by subtracting the sum of the readings of the three capillaries of the respective solution in the empty chamber from the equivalent values in the experimental chamber. Consumption per fly was obtained by dividing sucrose or yeast extract total consumption by the number of living flies at the end of the assay.

### flyPAD assays

flyPAD assays were performed as described in [67]. For food choice experiments, single flies in different dietary conditions were tested in arenas that contained two kinds of food patches: 10% yeast and 20 mM sucrose, each mixed with 1% agarose.

To measure the phagostimulatory power of bacteria (Fig 5E), we used a flyPAD setup that had never been exposed to yeast. All tested flies were deprived from amino acids using HM–AAs. For the flyPAD assays, one feeding well per arena was filled with HM without sucrose, either intact media (holidic AA medium) or media supplemented with the bacterial mixture (holidic AA medium with five bacteria) (in HM: *L*. *plantarum*^*WJL*^ 1.02 x 10^2^ CFU, *L*. *brevis*^*EW*^ 8.49 CFU, *A*. *pomorum* 1.52 x 10^2^ CFU, *C*. *intestini*^*A911T*^ 1.45 x 10^2^ CFU, and *E*. *faecalis* 1.77 x 10^2^ CFU). These media were prepared by adding agarose (1%) as a gelling agent together with cholesterol after autoclaving. Media were prepared on the experimental day and maintained at 30°C in a heat block. Preparation of the control and bacterial mixture pellets were performed as described above and directly resuspended in the HM without sucrose to generate holidic AA medium and holidic AA medium with five bacteria, respectively. Each medium was loaded into a single feeding well of the arena.

For all experiments, flies were individually transferred to flyPAD arenas by mouth aspiration and allowed to feed for 1 h at 25°C, 70% relative humidity. The total number of sips per animal over this hour was calculated using previously described flyPAD algorithms [67]. Noneating flies (defined as having fewer than two activity bouts during the assay) were excluded from the analysis.

### Egg-laying assays

Groups of 16 female and 5 male flies were briefly anesthetized using light CO_2_ exposure and transferred to apple juice agar plates (per liter, 250 ml apple juice, 19.5 g agar, 20 g sugar, and 10 ml nipagin [15% in ethanol]), where they were allowed to lay eggs for 24 h. Flies were then removed and counted and eggs were counted. Egg laying was calculated by dividing the number of eggs by the number of living females at the end of the assay.

### Total mRNA extraction, RT-PCR, and quantitative real-time PCR

Flies used for mRNA extraction were snap frozen in dry ice and kept at –80°C until used. Behavioral assays were performed in parallel to confirm that sibling flies presented the expected feeding phenotype. mRNA was extracted from flies (three flies per condition) using the following procedure: flies were grinded and homogenized for 20 s (using pestles #Z359947, Sigma) in 100 μl of PureZOL (#732–6890, Bio-Rad). 250 μl of PureZOL was further added and mixed by pipetting and incubated at RT for 10 min. Finally, 350 μl of 100% ethanol was added, and the samples were mixed and transferred to a Zymo column (Direct-zol RNA MicroPrep #R2062, Zymo research). The manufacturer’s instructions were followed to purify the mRNA (including DNAse treatment), and samples were eluted in 15 μl of distilled RNase/DNase-free water. The concentration of the total mRNA samples was determined by performing a spectrophotometer scan in the UV region. Total RNA (1 μg) was reverse transcribed (RT) using the iScript Reverse Transcription Supermix for RT-PCR kit (#170–8840 Bio-Rad), following the manufacturer’s instructions. The expression of *Henna* was determined using real-time PCR. Each cDNA sample was amplified using SsoFast EvaGreen Supermix on the CFX96 Real-Time System (Bio-Rad). Briefly, the reaction conditions consisted of 1 μl of 1:10 diluted cDNA, 1 μl (10 μM) of each primer, 10 μl of supermix, and 7 μl of water. The cycle program consisted of enzyme activation at 95°C for 30 s, 39 cycles of denaturation at 95°C for 2 s, and annealing and extension for 5 s. The primers used in this reaction are listed in S4 Table. This experiment was performed using three experimental replicas and two technical replicas per genotype. Appropriate nontemplate controls were included in each 96-well PCR reaction, and dissociation analysis was performed at the end of each run to confirm the specificity of the reaction. Absolute levels of RNA were calculated from a standard curve and normalized to the internal controls (*Actin42A* and *RpL32*). The relative quantitation of each mRNA was performed using the comparative Ct method. Data processing was performed using Bio-rad CFX Manager 3.1 (Bio-Rad).

### Amino acid measurements in fly heads

500 females per condition were collected on the same day as behavioral assays and were snap frozen in dry ice. Flies were kept at –80°C until head preparation for amino acids measurements. Fly heads were separated from other body parts by vortexing the Eppendorf tubes and posteriorly passing the debris through 710-mm and 425-mm sieves (Retsch GmbH). Fly heads were counted before homogenization to ensure that the same number was used for all conditions. Heads were homogenized in 200 μl of 2.5% TCA and centrifuged for 10 min at top speed at 4°C. The supernatant was recovered and stored at 4°C for analysis. Amino acid quantification was performed by HPLC at a clinical laboratory (Joaquim Chaves Laboratories, PT). Amino acids were detected using AccQ.Tag (Waters, #176001235).

## Supporting information

S1 Fig Deprivation of amino acids specifically increases amino acid appetite. (A) Feeding assay in which flies were given the choice between the holidic medium lacking AAs (sucrose option) and the holidic medium lacking sucrose and one of the different nutrient classes. Animals were either kept on full holidic medium or holidic medium lacking AAs. (B) Feeding assay in which flies were given the choice between two options: 1) the holidic medium lacking amino acids (sucrose) and 2) the holidic medium lacking sucrose, lacking sucrose and all AAs, lacking sucrose and neAAs, or lacking sucrose and eAAs. Circles represent yeast preference in single assays, with line representing the median and whiskers the interquartile range. n = 12–15. (A) Significance was tested using the Kruskal-Wallis test followed by Dunn’s multiple comparison test or (B) the Mann Whitney test. Not significant (ns) p>0.05, *** p<0.001. Underlying data used in this Figure are provided in S1 Data.
(TIF)

S2 Fig *Henna* RNAi leads to specific gene knockdown, and the knockdown effect on food choice is Tyr and tissue specific. (A) *Henna* mRNA levels measured from whole flies and normalized to two internal controls (*Actin 42A* and *RpL32*). The columns represent the mean and the error bars the standard error of the mean. n = 6. (B) Feeding preference of *Henna* knockdown animals using three independent hairpins. Animals were kept on holidic medium lacking neAAs, holidic diet lacking neAAs with 1x Tyr added back, or holidic medium lacking neAAs with 1x Pro added back. n = 10. (C) Feeding preference of control and *Henna* (*Hn*) knockdown flies in different tissues upon removal of either all AAs or all neAAs. *Cg*-*Gal4* drives expression in fat body, *elav*-*Gal4* in neurons and *btl*-*Gal4* in trachea. n = 14–20. (B and C) Circles represent yeast preference in single assays, with line representing the median and whiskers the interquartile range. Significance was tested using the Kruskal-Wallis test followed by Dunn’s multiple comparison test. Not significant (ns) p>0.05, ** p<0.01, *** p<0.001. Underlying data used in this Figure are provided in S1 Data.
(TIF)

S3 Fig Levels of internal microbes in non-axenic flies. The internal load of bacteria inside flies was calculated as CFU/fly after bacterial colony count on LB (A), Mannitol (B), or MRS (C) media which sustain the growth of different bacterial species as indicated in the title of each graph. The load of bacteria was assessed for flies kept on holidic medium without His and without (empty columns) or with (filled columns) pretreatment with the commensal bacteria mix. Flies used to generate data in Figs 1, 2, 3, 4, 5E, 7, S1, S2, S4, S5A and S6 were treated using this or very similar rearing protocols. The columns represent the mean and the error bars, the standard error of the mean of 3 replicates from 2 independent experiments. Filled black circles represent pretreatment with the bacteria mix. Open circles represent no pretreatment with bacteria. AA deprivation is indicated as –His. Significance was tested using the unpaired t-test. * p<0.05, ** p<0.01. Underlying data used in this Figure are provided in S1 Data.
(TIF)

S4 Fig Commensal bacteria can reduce the protein appetite induced by dietary removal of any eAA. Feeding preference of animals kept either on holidic medium, or holidic medium lacking one of the 10 eAAs with or without pretreatment with 5 bacteria commensal mix. Data on the different graphs were collected on two independent days. Circles represent yeast preference in single assays, with line representing the median and whiskers the interquartile range. Filled circles represent assays in which flies had been pretreated with the 5 bacteria mix. n = 18–20. Significance was tested using the Kruskal-Wallis test followed by Dunn’s multiple comparison test, except for testing the effect of commensals, for which the Mann Whitney test was used. * p<0.05, ** p<0.01, *** p<0.001. Underlying data used in this Figure are provided in S1 Data.
(TIF)

S5 Fig A non-commensal bacterium does not reduce yeast preference and commensal bacteria also affect food choice on low-yeast diets. (A) Feeding preference of animals kept either on holidic medium, or holidic medium lacking His with or without pretreatment with the 5 commensal bacteria mix or *E*. *coli*. Circles represent yeast preference in single assays, with line representing the median and whiskers the interquartile range. (B) Feeding preference of animals kept on medium with different concentrations of yeast and with or without pretreatment with the 5 commensal bacteria mix. Circles represent means and error bars represent the standard error of the mean. (A and B) Filled circles represent assays in which flies had been pretreated with the bacteria mix. n = 20. Significance was tested using the One-way analysis of variance test followed by Bonferroni’s multiple comparison test in (A) and using the Mann Whitney test in (B). Not significant (ns) p>0.05, * p<0.05, ** p<0.01, *** p<0.001. Underlying data used in this Figure are provided in S1 Data.
(TIF)

S6 Fig Commensal bacteria reduce yeast preference in *Henna* knockdown flies upon eAA deprivation. Feeding preference of control and *Henna* knockdown animals kept on holidic medium, or holidic medium lacking His with or without pretreatment with 5 commensal bacteria mix. n = 20. Circles represent yeast preference in single assays, with line representing the median and whiskers the interquartile range. Filled circles represent assays in which flies had been pretreated with the bacteria mix. Significance was tested using the Mann Whitney test followed by Bonferroni correction. * p<0.05, ** p<0.01, *** p<0.001. Underlying data used in this Figure are provided in S1 Data.
(TIF)

S7 Fig *A*. *pomorum* and *Lactobacilli* are sufficient to reduce the yeast appetite induced by dietary removal of isoleucine. Numbers of sips on yeast as measured using the flyPAD of axenic flies pre-fed complete holidic medium or holidic medium without Ile and pretreated with different bacterial mixes. Filled black circles represent complete holidic medium or presence of specific bacteria in the pretreatment mix. Open black circles represent absence of specific bacteria in the pretreatment mix. Ile deprivation is indicated as -Ile. Boxes represent upper and lower quartiles with median. n = 25–55. Significance was tested using the Kruskal-Wallis test followed by Dunn’s multiple comparison test. *** p<0.001. Underlying data used in this Figure are provided in S1 Data.
(TIF)

S8 Fig Diagram depicting the chronology of the dietary and microbial manipulations of flies used in experiments. (TIF)

S1 Table Detailed genotypes of flies used in this study. (DOCX)

S2 Table Recipe for generation of HM used in this study. Gray fields indicate manipulated nutrients.
(DOCX)

S3 Table Detailed composition of amino acid solutions for HM used in this study. (DOCX)

S4 Table Nucleotide sequence of primers used for qRT-PCR. (DOCX)

S1 Data Raw data used for generation of all figures. (XLSX)

## Abbreviations

AA: amino acid
*Ap*: *Acetobacter pomorum*
Arg: arginine
CAFE: capillary feeder assay
eAA: essential amino acid
His: histidine
Ile: isoleucine
*Lb*: *Lactobacillus brevis*
*Lp*: *Lactobacillus plantarum*
neAA: nonessential amino acid
ns: not significant
Phe: phenylalanine
Tyr: tyrosine
Val: valine
