# Supplementary figures and images for: Commensal bacteria and essential amino acids control food choice behavior and reproduction
Source: PLoS Biol. 2017 Apr 25;15(4):e2000862. doi: 10.1371/journal.pbio.2000862 (PMC5404834; doi:10.1371/journal.pbio.2000862)

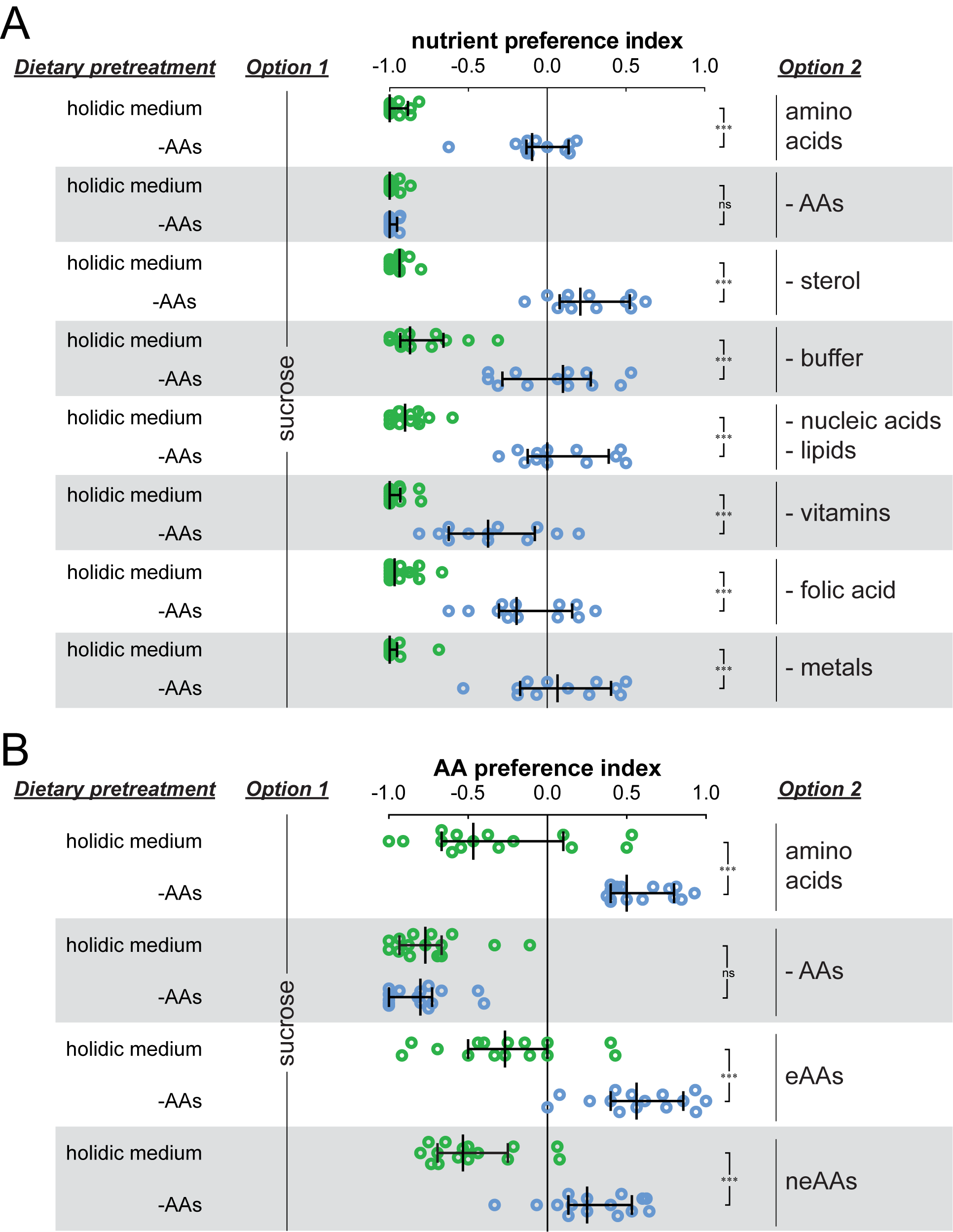

Supplement: S1 Fig — (A) Feeding assay in which flies were given the choice between the holidic medium lacking AAs (sucrose option) and the holidic medium lacking sucrose and one of the different nutrient classes. Animals were either kept on full holidic medium or holidic medium lacking AAs. (B) Feeding assay in which flies were given the choice between two options: 1) the holidic medium lacking amino acids (sucrose) and 2) the holidic medium lacking sucrose, lacking sucrose and all AAs, lacking sucrose and neAAs, or lacking sucrose and eAAs. Circles represent yeast preference in single assays, with line representing the median and whiskers the interquartile range. n = 12–15. (A) Significance was tested using the Kruskal-Wallis test followed by Dunn’s multiple comparison test or (B) the Mann Whitney test. Not significant (ns) p>0.05, *** p<0.001. Underlying data used in this Figure are provided in S1 Data. (TIF) [file pbio.2000862.s001.tif]

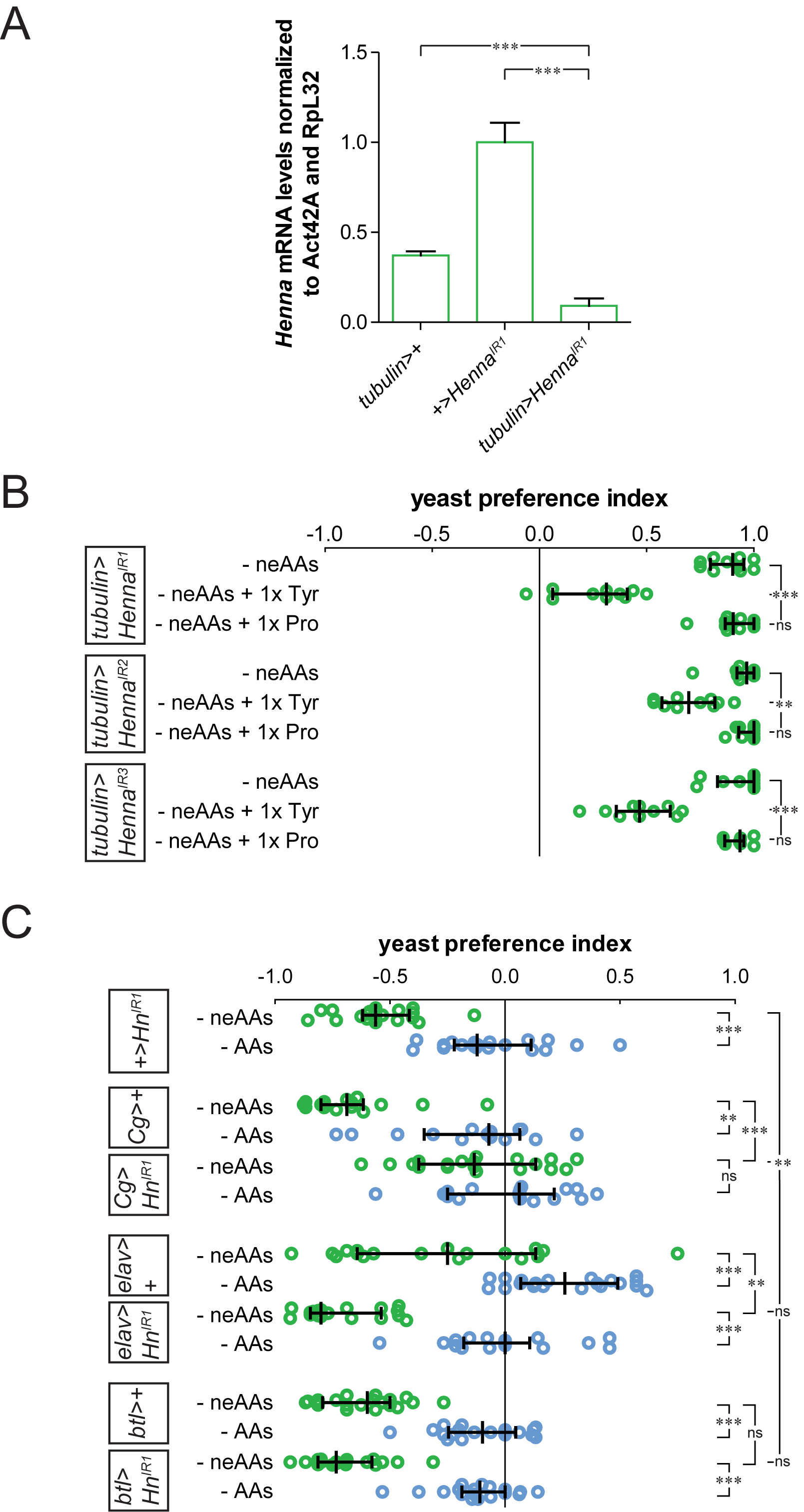

Supplement: S2 Fig — (A) Henna mRNA levels measured from whole flies and normalized to two internal controls (Actin 42A and RpL32). The columns represent the mean and the error bars the standard error of the mean. n = 6. (B) Feeding preference of Henna knockdown animals using three independent hairpins. Animals were kept on holidic medium lacking neAAs, holidic diet lacking neAAs with 1x Tyr added back, or holidic medium lacking neAAs with 1x Pro added back. n = 10. (C) Feeding preference of control and Henna (Hn) knockdown flies in different tissues upon removal of either all AAs or all neAAs. Cg-Gal4 drives expression in fat body, elav-Gal4 in neurons and btl-Gal4 in trachea. n = 14–20. (B and C) Circles represent yeast preference in single assays, with line representing the median and whiskers the interquartile range. Significance was tested using the Kruskal-Wallis test followed by Dunn’s multiple comparison test. Not significant (ns) p>0.05, ** p<0.01, *** p<0.001. Underlying data used in this Figure are provided in S1 Data. (TIF) [file pbio.2000862.s002.tif]

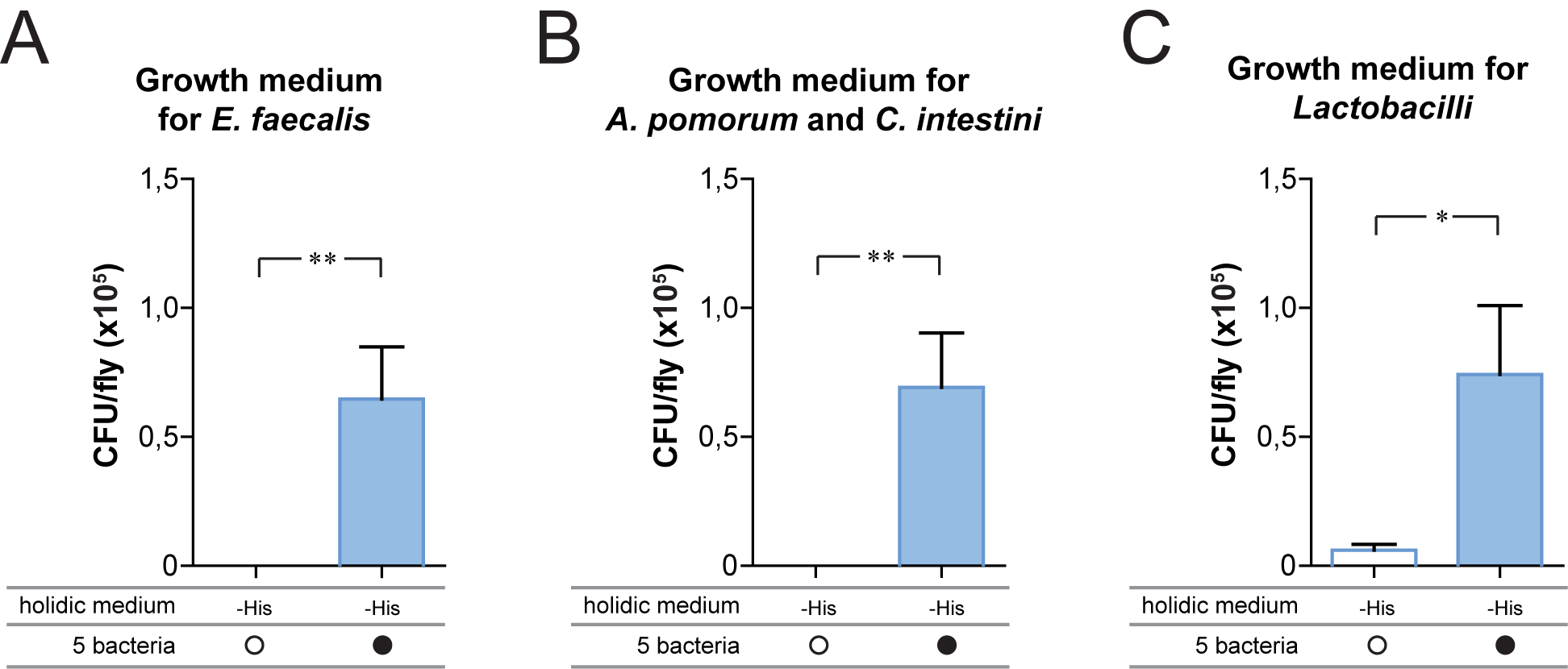

Supplement: S3 Fig — The internal load of bacteria inside flies was calculated as CFU/fly after bacterial colony count on LB (A), Mannitol (B), or MRS (C) media which sustain the growth of different bacterial species as indicated in the title of each graph. The load of bacteria was assessed for flies kept on holidic medium without His and without (empty columns) or with (filled columns) pretreatment with the commensal bacteria mix. Flies used to generate data in Figs 1, 2, 3, 4, 5E, 7, S1, S2, S4, S5A and S6 were treated using this or very similar rearing protocols. The columns represent the mean and the error bars, the standard error of the mean of 3 replicates from 2 independent experiments. Filled black circles represent pretreatment with the bacteria mix. Open circles represent no pretreatment with bacteria. AA deprivation is indicated as –His. Significance was tested using the unpaired t-test. * p<0.05, ** p<0.01. Underlying data used in this Figure are provided in S1 Data. (TIF) [file pbio.2000862.s003.tif]

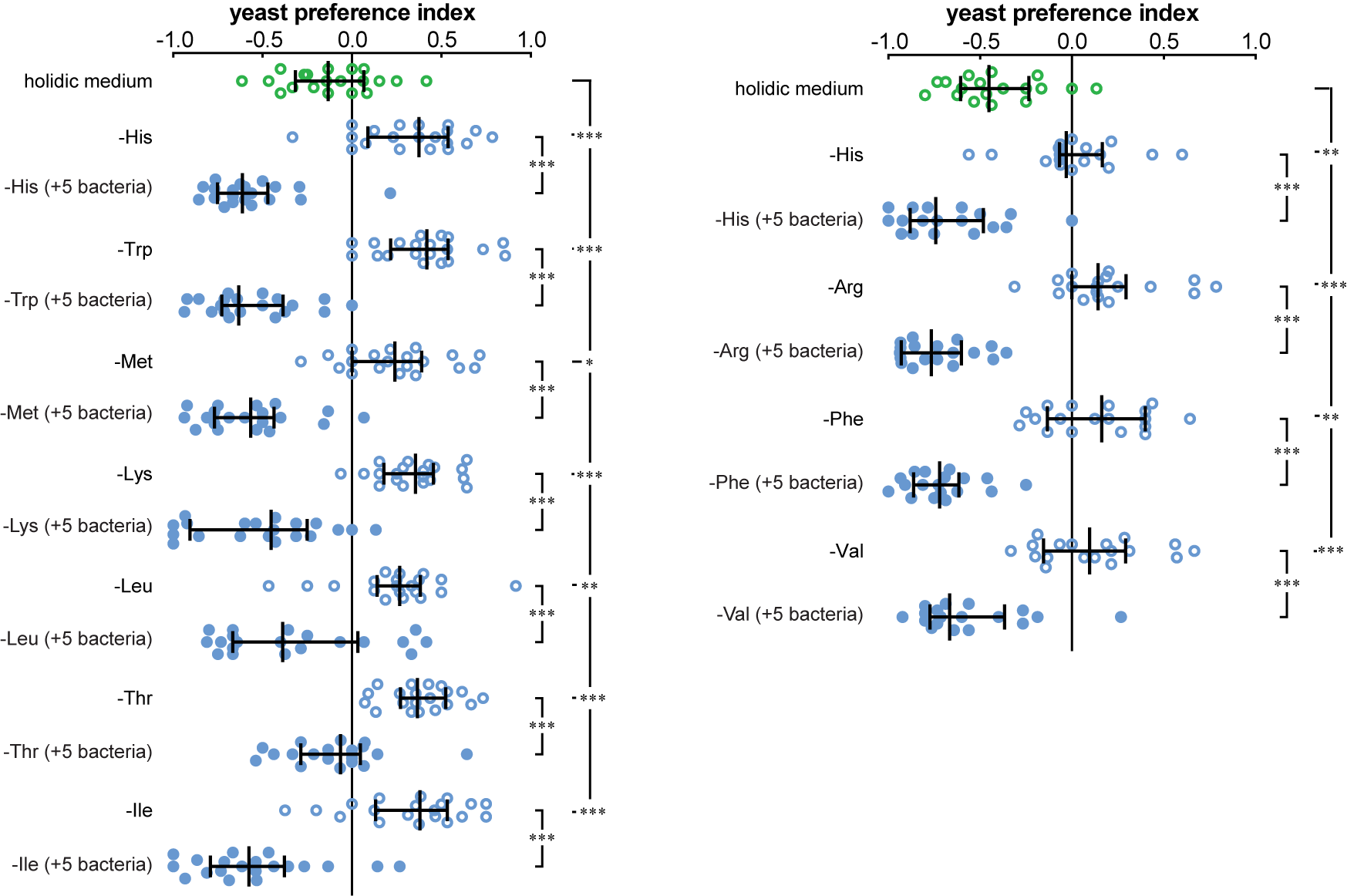

Supplement: S4 Fig — Feeding preference of animals kept either on holidic medium, or holidic medium lacking one of the 10 eAAs with or without pretreatment with 5 bacteria commensal mix. Data on the different graphs were collected on two independent days. Circles represent yeast preference in single assays, with line representing the median and whiskers the interquartile range. Filled circles represent assays in which flies had been pretreated with the 5 bacteria mix. n = 18–20. Significance was tested using the Kruskal-Wallis test followed by Dunn’s multiple comparison test, except for testing the effect of commensals, for which the Mann Whitney test was used. * p<0.05, ** p<0.01, *** p<0.001. Underlying data used in this Figure are provided in S1 Data. (TIF) [file pbio.2000862.s004.tif]

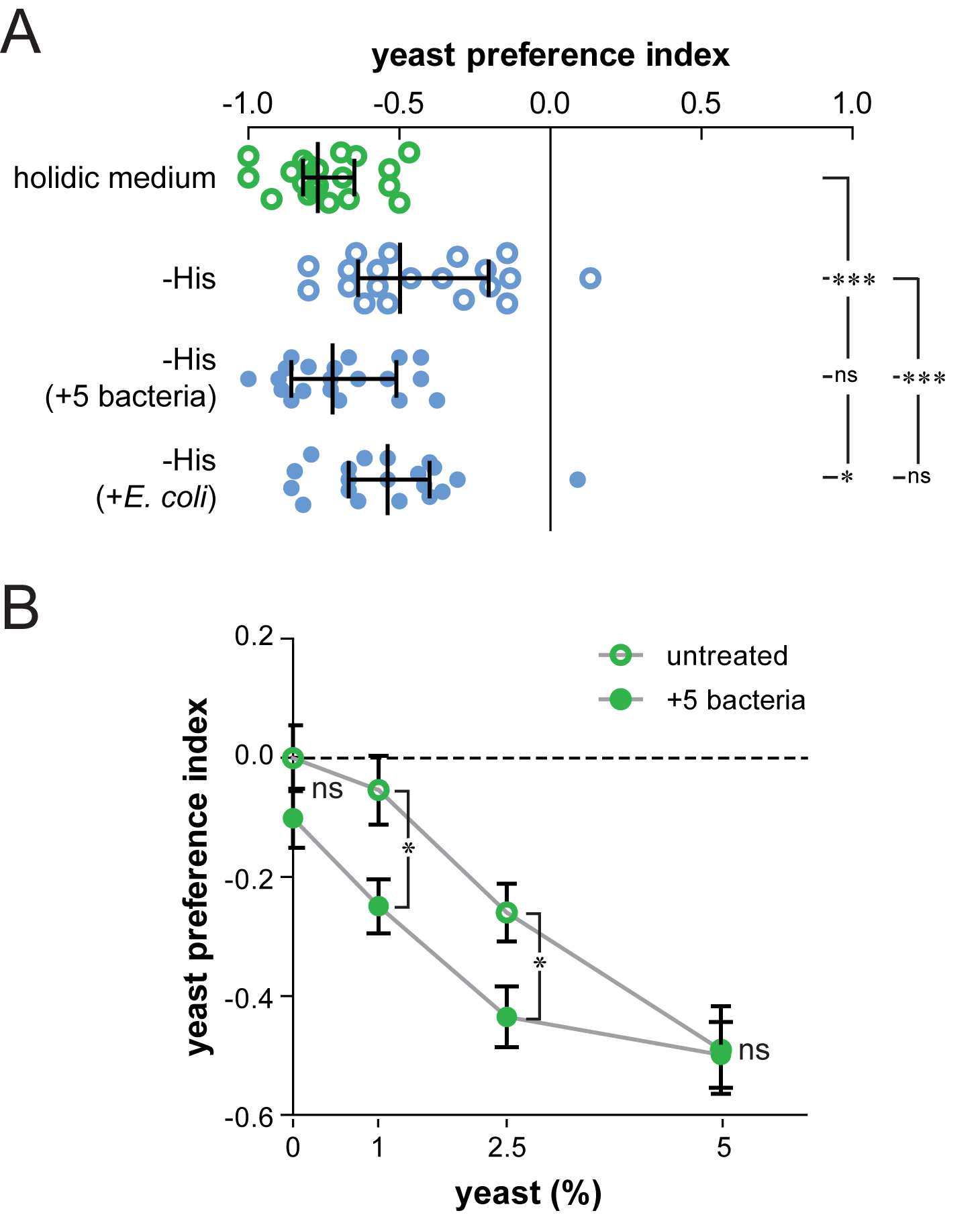

Supplement: S5 Fig — (A) Feeding preference of animals kept either on holidic medium, or holidic medium lacking His with or without pretreatment with the 5 commensal bacteria mix or E. coli. Circles represent yeast preference in single assays, with line representing the median and whiskers the interquartile range. (B) Feeding preference of animals kept on medium with different concentrations of yeast and with or without pretreatment with the 5 commensal bacteria mix. Circles represent means and error bars represent the standard error of the mean. (A and B) Filled circles represent assays in which flies had been pretreated with the bacteria mix. n = 20. Significance was tested using the One-way analysis of variance test followed by Bonferroni’s multiple comparison test in (A) and using the Mann Whitney test in (B). Not significant (ns) p>0.05, * p<0.05, ** p<0.01, *** p<0.001. Underlying data used in this Figure are provided in S1 Data. (TIF) [file pbio.2000862.s005.tif]

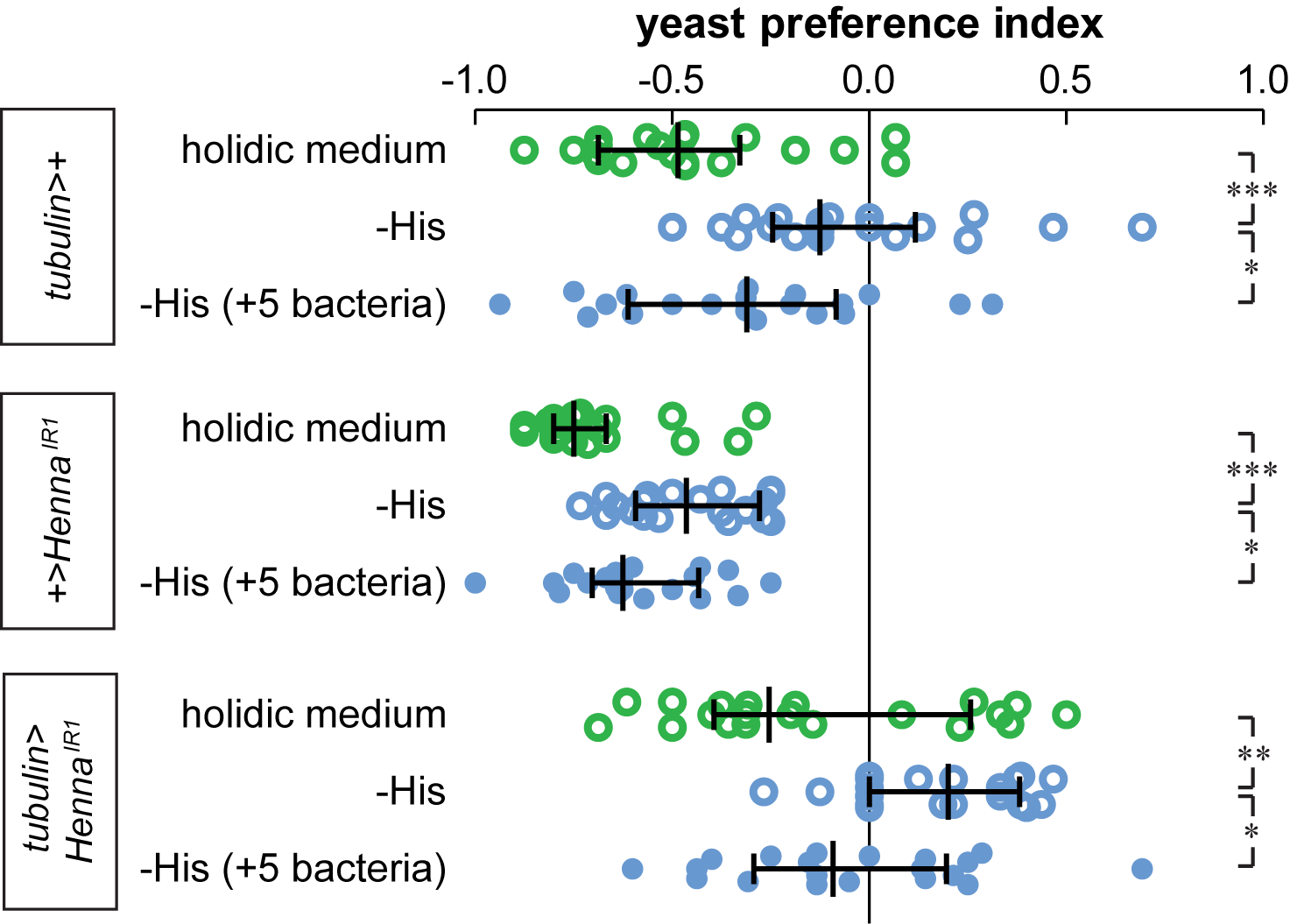

Supplement: S6 Fig — Feeding preference of control and Henna knockdown animals kept on holidic medium, or holidic medium lacking His with or without pretreatment with 5 commensal bacteria mix. n = 20. Circles represent yeast preference in single assays, with line representing the median and whiskers the interquartile range. Filled circles represent assays in which flies had been pretreated with the bacteria mix. Significance was tested using the Mann Whitney test followed by Bonferroni correction. * p<0.05, ** p<0.01, *** p<0.001. Underlying data used in this Figure are provided in S1 Data. (TIF) [file pbio.2000862.s006.tif]

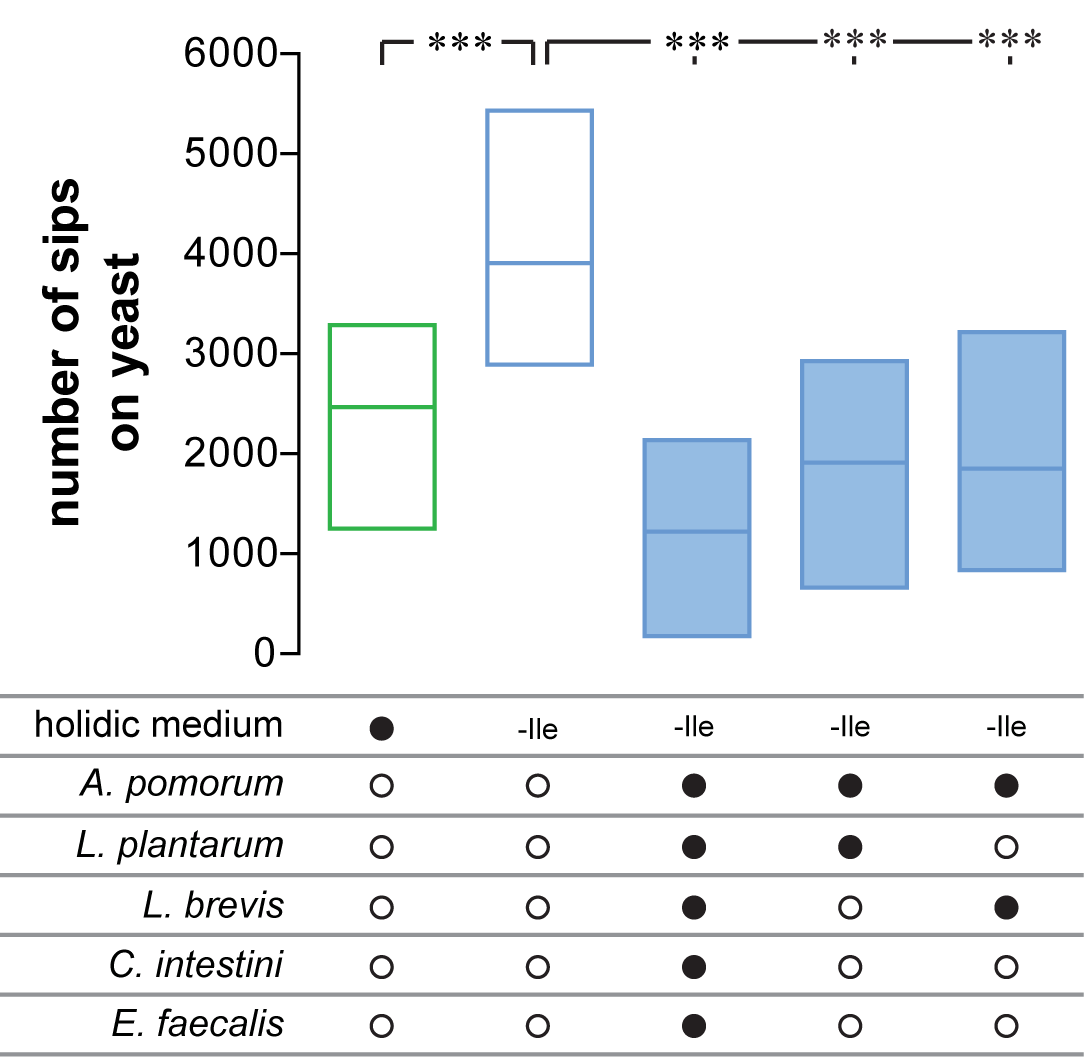

Supplement: S7 Fig — Numbers of sips on yeast as measured using the flyPAD of axenic flies pre-fed complete holidic medium or holidic medium without Ile and pretreated with different bacterial mixes. Filled black circles represent complete holidic medium or presence of specific bacteria in the pretreatment mix. Open black circles represent absence of specific bacteria in the pretreatment mix. Ile deprivation is indicated as -Ile. Boxes represent upper and lower quartiles with median. n = 25–55. Significance was tested using the Kruskal-Wallis test followed by Dunn’s multiple comparison test. *** p<0.001. Underlying data used in this Figure are provided in S1 Data. (TIF) [file pbio.2000862.s007.tif]

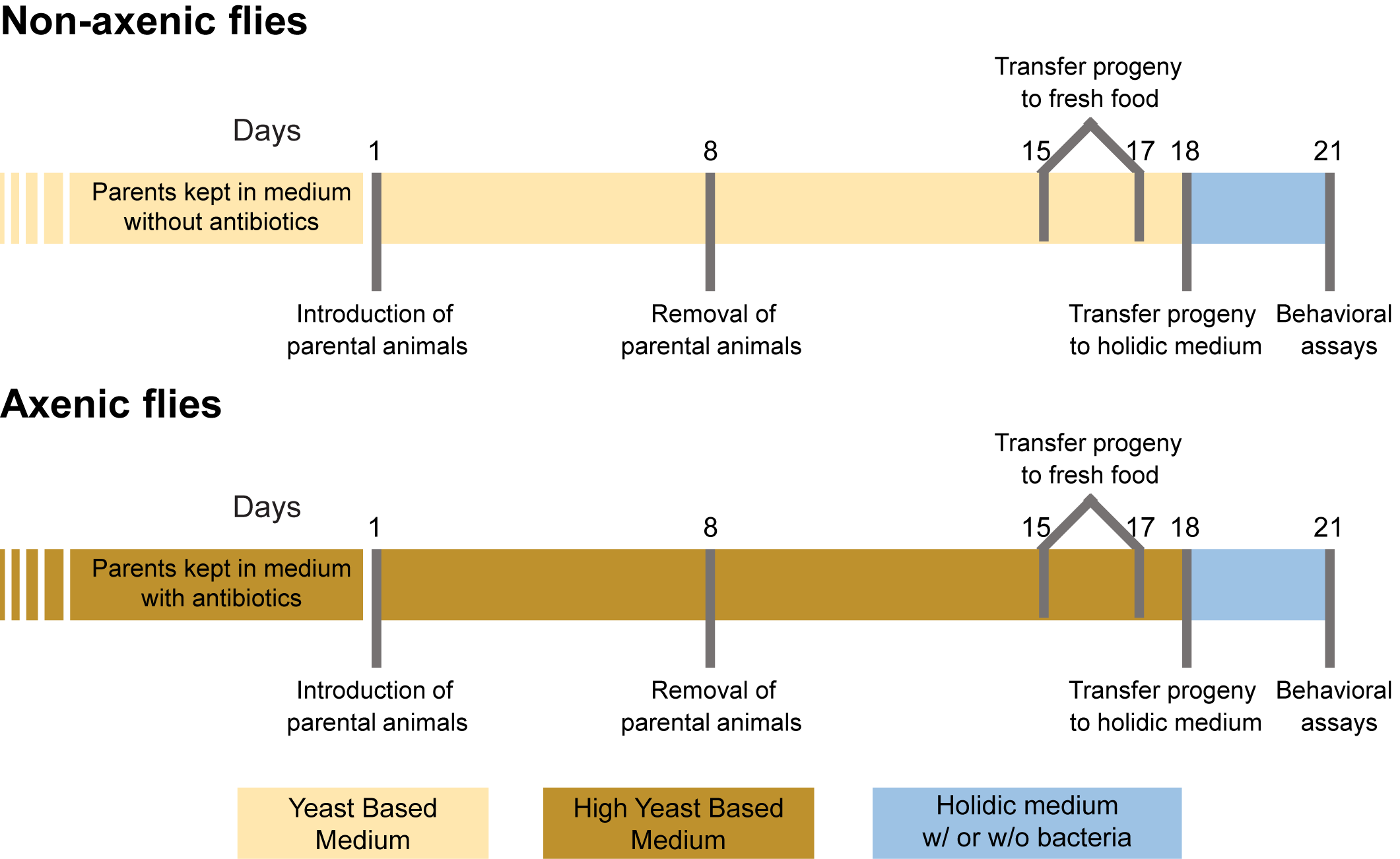

Supplement: S8 Fig — (TIF) [file pbio.2000862.s008.tif]
