# Supplementary material for: Commensal bacteria and essential amino acids control food choice behavior and reproduction
Source: PLoS Biol. 2017 Apr 25;15(4):e2000862. doi: 10.1371/journal.pbio.2000862 (PMC5404834; doi:10.1371/journal.pbio.2000862)
Supplement: S1 Table — (DOCX) [file pbio.2000862.s009.docx]

| **Referred to as** | **Detailed genotype** |
| --- | --- |
| control background | *w^1118^* |
| *tubulin>+* | *y* / +, w* / w^1118^ ; ; P{tubP-GAL4} / +* |
| *Cg>+* | *w^1118^/ +; P{Cg-GAL4.A}2 / +;* |
| *elav>+* | *w^1118^, P{w[+mC]=UAS-Dcr-2} / +; ; P{w[+mC]=GAL4-elav.L}3 / +* |
| *btl>+* | *w* / w^1118^; ; P{w+, btl-GAL4} / +* |
| *+>Henna^IR1^* | *w^1118^; ; P{GD35240}^v11574^ / +* |
| *tubulin>Henna^IR1^* | *y* / +, w* / w^1118^; ; P{tubP-GAL4} / P{GD35240}^v11574^* |
| *tubulin>Henna^IR2^* | *y* / +, w* / w^1118^; ; P{tubP-GAL4} / P{NIG.7399R}3* |
| *tubulin>Henna^IR3^* | *y* / +, w* / +, v^1^ / +; ; P{tubP-GAL4} / P{TRiP.JF02261}attP2* |
| *Cg>Henna^IR1^* | *w^1118^; P{Cg-GAL4.A}2 / +; P{GD35240}^v115747^ / +* |
| *elav>Henna^IR1^* | *w^1118^, P{w[+mC]=UAS-Dcr-2} / +; ; P{w[+mC]=GAL4-elav.L}3 / P{GD35240}^v11574^* |
| *btl>Henna^IR1^* | *w* / w^1118^; ; P{w+, btl-GAL4} / P{GD35240}^v11574^* |
