# Supplementary material for: Commensal bacteria and essential amino acids control food choice behavior and reproduction
Source: PLoS Biol. 2017 Apr 25;15(4):e2000862. doi: 10.1371/journal.pbio.2000862 (PMC5404834; doi:10.1371/journal.pbio.2000862)
Supplement: S2 Table — Gray fields indicate manipulated nutrients. (DOCX) [file pbio.2000862.s010.docx]

| **Referred to as** | holidic medium (HUNTaa) | -all AAs | -eAAs | -neAAs | -single eAA | -neAAs + 1xTyr (or 2xTyr) | -neAAs + 1xPro |
| --- | --- | --- | --- | --- | --- | --- | --- |
| Essential amino acids | 60,51 ml | 0 ml | 0 ml | 140,45 ml(a) | 140,45 ml(a,b) | 140,45 ml(a) | 140,45 ml(a) |
| L-isoleucine | 1,82 g | 0 g | 0 g | 4,21 g(a) | 4,21 or 0 g(a,c) | 4,21 g(a) | 4,21 g(a) |
| L-leucine | 1,21 g | 0 g | 0 g | 2,81 g(a) | 2,81 or 0 g(a,c) | 2,81 g(a) | 2,81 g(a) |
| Non-essential amino acids | 60,51 ml | 0 ml | 97,52 ml(a) | 0 ml | 0 ml | 0 ml | 0,27 g proline(d) |
| L-glutamate | 15,13 ml | 0 ml | 24,38 ml(a) | 35,11 ml(a) | 35,11 ml(a) | 35,11 ml(a) | 35,11 ml(a) |
| L-tyrosine | 0,42 g | 0 g | 1,61 g(a) | 0 g | 0 g | 0,42 g (1x) or 0,84 g (2x) | 0 g |
| L-cysteine (HCl) | n/a(e) | n/a(e) | n/a(e) | n/a(e) | n/a(e) | n/a(e) | n/a(e) |
| Cholesterol | 15 ml | 15 ml | 15 ml | 15 ml | 15 ml | 15 ml | 15 ml |
| CaCl2 | 1 ml | 1 ml | 1 ml | 1 ml | 1 ml | 1 ml | 1 ml |
| MgSO4 | 1 ml | 1 ml | 1 ml | 1 ml | 1 ml | 1 ml | 1 ml |
| CuSO4 | 1 ml | 1 ml | 1 ml | 1 ml | 1 ml | 1 ml | 1 ml |
| FeSO4 | 1 ml | 1 ml | 1 ml | 1 ml | 1 ml | 1 ml | 1 ml |
| MnCl2 | 1 ml | 1 ml | 1 ml | 1 ml | 1 ml | 1 ml | 1 ml |
| ZnSO4 | 1 ml | 1 ml | 1 ml | 1 ml | 1 ml | 1 ml | 1 ml |
| Nucleic acids & Lipids | 8 ml | 8 ml | 8 ml | 8 ml | 8 ml | 8 ml | 8 ml |
| Vitamins | 14 ml | 14 ml | 14 ml | 14 ml | 14 ml | 14 ml | 14 ml |
| Folic acid | 1 ml | 1 ml | 1 ml | 1 ml | 1 ml | 1 ml | 1 ml |
| Acetic acid buffer | 100 ml | 100 ml | 100 ml | 100 ml | 100 ml | 100 ml | 100 ml |
| Sucrose | 17,12 g | 17,12 g | 17,12 g | 17,12 g | 17,12 g | 17,12 g | 17,12 g |
| Agar | 20 g | 20 g | 20 g | 20 g | 20 g | 20 g | 20 g |
| Milli Q H2O | Adjust to 1 L | Adjust to 1 L | Adjust to 1 L | Adjust to 1 L(f) | Adjust to 1 L | Adjust to 1 L | Adjust to 1 L |

(a) The amount of these nutrients was increased to adjust concentration of biological active nitrogen to 197,9 mM as in the complete HM. In diets where neAAs were removed, L-glutamate was still added.
(b) A complete essential AAs solution or an essential AA drop-out solution lacking L-arg, L-met, L-val, L-phe, L-his, L-lys, L-thr or L-trp were used.
(c) L-ile or L-leu were omitted to obtain HM lacking these AAs.
(d) The amount of Proline used corresponds to the biological active nitrogen equivalent to 0,42 g of L-tyr (2,32 mmol).

(e) L-cysteine HCl was added in a separate solution only in the HM with improved AA composition.

(f) In the absence of medium preservatives, Cg-Gal4 flies were not healthy and therefore 6 ml of propionic acid and 15 ml of nipagin (100 g/l in 95% EtOH) were added.

| **Referred to as** | -single eAA (g) | -folic acid | -metals | -nc.ac. & lipids | -sterol | -vitamins | holidic medium (improved AA composition) (h) |
| --- | --- | --- | --- | --- | --- | --- | --- |
| Essential amino acids | 60,51 ml(b) | 60,51 ml | 60,51 ml | 60,51 ml | 60,51 ml | 60,51 ml | 60,51 ml(i) |
| L-isoleucine | 1,82 or 0 g(c) | 1,82 g | 1,82 g | 1,82 g | 1,82 g | 1,82 g | 1,12 g or 0 g(j) |
| L-leucine | 1,21 or 0 g(c) | 1,21 g | 1,21 g | 1,21 g | 1,21 g | 1,21 g | 2,03 g |
| Non-essential amino acids | 60,51 ml | 60,51 ml | 60,51 ml | 60,51 ml | 60,51 ml | 60,51 ml | 60,51 ml |
| L-glutamate | 15,13 ml | 15,13 ml | 15,13 ml | 15,13 ml | 15,13 ml | 15,13 ml | 15,19 ml |
| L-tyrosine | 0,42 g | 0,42 g | 0,42 g | 0,42 g | 0,42 g | 0,42 g | 0,93 g |
| L-cysteine (HCl) | n/a(e) | n/a(e) | n/a(e) | n/a(e) | n/a(e) | n/a(e) | 6,83 ml(e) |
| Cholesterol | 15 ml | 15 ml | 15 ml | 15 ml | 0 ml | 15 ml | 15 ml |
| CaCl2 | 1 ml | 1 ml | 0 ml | 1 ml | 1 ml | 1 ml | 1 ml |
| MgSO4 | 1 ml | 1 ml | 0 ml | 1 ml | 1 ml | 1 ml | 1 ml |
| CuSO4 | 1 ml | 1 ml | 0 ml | 1 ml | 1 ml | 1 ml | 1 ml |
| FeSO4 | 1 ml | 1 ml | 0 ml | 1 ml | 1 ml | 1 ml | 1 ml |
| MnCl2 | 1 ml | 1 ml | 0 ml | 1 ml | 1 ml | 1 ml | 1 ml |
| ZnSO4 | 1 ml | 1 ml | 0 ml | 1 ml | 1 ml | 1 ml | 1 ml |
| Nucleic acids & Lipids | 8 ml | 8 ml | 8 ml | 0 ml | 8 ml | 8 ml | 8 ml |
| Vitamins | 14 ml | 14 ml | 14 ml | 14 ml | 14 ml | 0 ml | 21 ml |
| Folic acid | 1 ml | 0 ml | 1 ml | 1 ml | 1 ml | 1 ml | 1 ml |
| Acetic acid buffer | 100 ml | 100 ml | 100 ml | 100 ml | 100 ml | 100 ml | 100 ml |
| Sucrose | 17,12 g | 17,12 g | 17,12 g | 17,12 g | 17,12 g | 17,12 g | 17,12 g |
| Agar | 20 g | 20 g | 20 g | 20 g | 20 g | 20 g | 20 g |
| Milli Q H2O | Adjust to 1 L | Adjust to 1 L | Adjust to 1 L | Adjust to 1 L | Adjust to 1 L | Adjust to 1 L | Adjust to 1 L |

(b) A complete essential AAs solution or an essential AA drop-out solution lacking L-arg, L-met, L-val, L-phe, L-his, L-lys, L-thr or L-trp were used.
(c) L-ile or L-leu were omitted to obtain HM lacking these AAs.

(e) L-cysteine HCl was added in a separate solution only in the HM (with improved AA composition).

(g) Used for experiments in Figures 4, 7, S4, S5 and S6.

(h) Used for experiments in Figures 5, 6, and S7.

(i) A complete essential AAs solution or an essential AA drop-out solution lacking L-his were used.

(j) L-ile was omitted to obtain HM lacking this AA.
