# Supplementary material for: Commensal bacteria and essential amino acids control food choice behavior and reproduction
Source: PLoS Biol. 2017 Apr 25;15(4):e2000862. doi: 10.1371/journal.pbio.2000862 (PMC5404834; doi:10.1371/journal.pbio.2000862)
Supplement: S3 Table — (DOCX) [file pbio.2000862.s011.docx]

| **Referred to as** |  | holidic medium (HUNTaa) (b) | holidic medium (improved AA composition) (a) |
| --- | --- | --- | --- |
| Essential amino acid stock solution (1L) | L-arginine | 8 g | 26,95 g |
|  | L-histidine | 10 g | 10,8 g |
|  | L-lysine (HCl) | 19 g | 22,5 g |
|  | L-methionine | 8 g | 9,95 g |
|  | L-phenylalanine | 13 g | 16,65 g |
|  | L-threonine | 20 g | 18,3 g |
|  | L-tryptophan | 5 g | 5,3 g |
|  | L-valine | 28 g | 19,85 g |
| Non-essential amino acid stock solution (1L) | L-alanine | 35 g | 18,2 g |
|  | L-asparagine | 17 g | 17 g |
|  | L-aspartic acid | 17 g | 19,35 g |
|  | L-cysteine (HCl) | 0,5 g | n/a |
|  | L-glutamine | 25 g | 18,55 g |
|  | Glycine | 32 g | 12,7 g |
|  | L-proline | 15 g | 16,15 g |
|  | L-serine | 19 g | 22,8 g |
| L-glutamate stock solution (1L) | L-glutamate | 100 g | 100g |
| L-cysteine (HCl) stock solution (1L) | L-cysteine (HCl) | n/a | 50 g |

(a) Used for experiments in Figures 5, 6, and S7.

(b) Used for experiments in all remaining Figures.
