# Supplementary material for: Commensal bacteria and essential amino acids control food choice behavior and reproduction
Source: PLoS Biol. 2017 Apr 25;15(4):e2000862. doi: 10.1371/journal.pbio.2000862 (PMC5404834; doi:10.1371/journal.pbio.2000862)
Supplement: S4 Table — (DOCX) [file pbio.2000862.s012.docx]

| **Referred to as** | **Primer sequence** |
| --- | --- |
| *Henna Fwd* | 5' ACAACCACGTCTTTCCCTTG 3' |
| *Henna Rev* | 5' GCAGAGTGAAACCTGTGCAA 3' |
| *Actin42A Fwd* | 5' CAGGCGGTGCTTTCTCTCTA 3' |
| *Actin42A Rev* | 5' AGCTGTAACCGCGCTCAGTA 3' |
| *RpL32 Fwd* | 5' GCCCAAGATCGTGAAGAAGC 3' |
| *RpL32 Rev* | 5' GCACTCTGTTGTCGATACCCTTG 3' |
